# Food Safety and Nutritional Aspects of Plant‐Based Dairy and Meat Alternatives: A Review

**DOI:** 10.1002/fsn3.72062

**Published:** 2026-07-07

**Authors:** Elina Sohlberg, Petra Pasonen, Hanna‐Leena Alakomi, Johanna Suomi

**Affiliations:** ^1^ Technical Research Centre of Finland VTT Ltd Espoo Finland; ^2^ Risk Assessment Unit Finnish Food Authority Helsinki Finland

**Keywords:** contaminants, dairy and meat imitates, foodborne microbes, food safety, plant‐based meat alternatives, plant‐based milk alternatives

## Abstract

The consumption and availability of plant‐based dairy and milk alternatives are increasing. These products can contain chemical and microbiological hazards, which are not prevalent in animal‐based foods. On the other hand, the occurrence of foodborne hazards such as contaminants or pathogens found in animal‐based foods can also be lower in plant‐based alternatives. Currently, the European legislation has few limits to foodborne hazards in dairy and milk alternatives. This review aims to present the relevant chemical and microbiological foodborne hazards in different types of dairy and meat alternatives, discuss their potential for health risk to consumers and risk management options. Nutritional aspects are discussed briefly from the point of view of consumers' health.

## Graphical abstract

Plant‐based dairy and meat alternatives (PBDA and PBMA) offer sustainability benefits but also introduce chemical and microbiological hazards to the diet. Key concerns include emerging mycotoxins, heavy metals, process contaminants, and heat‐resistant spore‐forming bacteria. Comprehensive surveillance and tailored risk management strategies are needed as consumption increases.

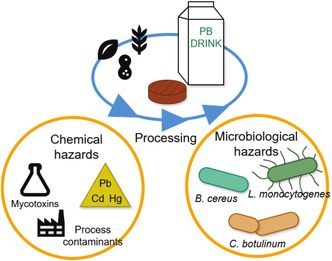

## 1 Introduction

The world population is increasing, and we need to feed over 10 billion people by 2050 (Arora et al. 2023). To succeed, we must increase global food production without compromising the ecosystem and address current issues such as greenhouse gas emissions, animal welfare, and land‐water usage that are caused by current meat production practices. To overcome the shortage of animal‐based foods and to achieve a reduction of meat protein usage in the diet, plants, insects, and microalgae are considered as a novel source of protein (Bakhsh et al. 2021). Increasing the consumption of plant‐based protein is important for environmental and health reasons, and it is also included in recommendations, such as the Nordic dietary recommendations and the European Green Development Program. The number of vegans, vegetarians, and particularly flexible herbivores (flexitarians), who occasionally also eat meat, is increasing (Ismail et al. 2020). As the number of vegetarians increases, the consumption of plant proteins and the selection of different plant protein products have also increased. According to Perez‐Cueto et al. (2024), 25% of Europeans surveyed reported they follow a vegetarian diet. The most common plant‐based diet was flexitarians, where individuals occasionally consume meat. Flexitarians comprised 16% of the respondents. Vegetarians who consume dairy products and eggs accounted for 4%, while pescatarians and vegans each represented approximately 2% of the respondents (Perez‐Cueto et al. 2024). Surveys have indicated that the proportion of vegetarians is highest among young adults and women. In these groups, even up to one‐third adhere to some vegetarian diet (Allès et al. 2017; Paslakis et al. 2020; Perez‐Cueto et al. 2024). The sales of plant‐based proteins have increased by 10% over the past 5 years (Innova 2024). Future forecasts suggest that the plant‐based food market will continue to demonstrate substantial growth (Future Market Insights 2025).

Dairy products, meat and many of the raw materials of plant‐based dairy (PBDA) or meat alternatives (PBMA) have maximum levels for contaminants set in legislation ((EU) 2023/915). However, the commercially available plant‐based dairy or meat alternatives are not yet subject to such legislative limits, apart from inorganic arsenic in rice‐based drinks. Currently, EU legislation ((EC) No 2073/2005) does not set specific microbiological criteria for PBDAs or PBMAs. As their use is growing, legislation will eventually be updated to cover also these products. More information on the occurrence of foodborne hazards in PBDAs and PBMAs is needed to identify the range of contamination and possible occurrence differences by origin.

The raw materials of plant‐based dairy or meat alternatives can be contaminated by hazards that are generally not found in animal‐based products, for example, a wide variety of mycotoxins as well as spore‐forming microbes. Some hazards can be present with a higher occurrence than in animal‐based products, for example, arsenic and possibly process contaminants in some types of products. The opposite, that is, PBDAs and PBMAs having a lower amount or lesser occurrence of certain hazards than animal‐based products, can also be observed. In addition, PBDAs and PBMAs can differ from their animal‐based products in their nutritional aspects.

Nutritional differences between plant‐based and animal‐based products have been studied more widely than the food safety perspective. This review will focus on the food safety, including both chemical and microbiological foodborne hazards. Replacing milk and meat with plant‐based alternatives affects both nutritional intake and exposure to foodborne hazards. These differences may influence consumer health, particularly among individuals who consume large amounts of plant‐based products. For this reason, this review article also compares the nutritional composition and the occurrence of hazards in plant‐based alternatives relative to milk and meat. The nutritional aspects will be discussed briefly, focusing on the possible health effects for the consumer. Antinutritive compounds present in some raw materials of plant‐based alternatives, for example, lectins, are not included in this review.

Although allergens can be a significant individual level risk (e.g., milk allergy, soy allergy, tree nut allergy, alpha‐gal syndrome showing as allergy to red meat), this review focuses on population level risks. Therefore, allergens are only mentioned in passim. Several reviews have been published on the topic of allergenic potential of alternative proteins, for example, Jappe (2023) and Milana et al. (2025).

Many of the raw materials of the plant‐based alternatives originate from South America or Asia. The aim of this review is to focus on the European market and foods falling under the EU legislation, and therefore the hazard profiles of American or Asian products are not explicitly addressed.

## 2 Methods

### 2.1 Literature Search Strategy

This literature review is a narrative synthesis of the available scientific literature on the food safety and nutritional aspects of plant‐based dairy and meat alternatives. No formal systematic review protocol was applied.

The literature was identified through targeted searches in Scopus, Google Scholar, and Iris.ai, complemented by manual screening of reference lists of relevant review articles, recent original studies, and reports from food safety authorities.

Search terms focused on the main themes of the review and included combinations of keywords such as plant‐based dairy alternatives, plant‐based milk imitates, plant‐based meat alternatives, food safety, mycotoxins, heavy metals, processing contaminants, microbiological hazards, Bacillus, Listeria, Salmonella, and risk assessment. Boolean operators like (AND, OR) were used to enhance the search.

Publications were primarily limited to articles or reports written in English, Finnish, French, or German. Emphasis was placed on recent literature published within the last decade, while older key publications were included when necessary to provide background or contextual understanding. Examples of foodborne outbreaks were primarily selected from Europe, as the review aims to reflect the regulatory context of the European Union and current EU food safety legislation.

Studies were selected based on their relevance to food safety hazards, processing effects, or nutritional implications of plant‐based alternatives, as well as their scientific quality. Official reports from recognized authorities (e.g., EFSA and national food safety agencies) were also included to complement peer‐reviewed literature.

## 3 Raw Materials of PBDAs and PBMAs and Associated Food Safety Hazards

### 3.1 PBDA and PBMA Ingredients

Various raw materials are utilized in the production of PBDA and PBMAs. The most common ingredients include legumes, cereal grains, and nuts. Additionally, new emerging ingredients such as mycoproteins and microalgae are also being incorporated.

#### 3.1.1 Legume‐Based Ingredients

Soy is one of the most widely used ingredients in both PBDAs and PBMAs. From a nutritional perspective, soy provides high‐quality protein, dietary fiber, and bioactive isoflavones, which have been associated with beneficial effects on lipid metabolism and cardiovascular health (Thavamani et al. 2020; Bakhsh et al. 2021). However, soy is also a food allergen, which is an important consideration in plant‐based product formulation and food safety. Other legumes used in PBMAs include pea, lentil, lupine, chickpea, and various beans (Hill 2022). Legumes are generally rich in protein and dietary fiber, although some species may have limitations in essential amino acid composition, such as methionine and cysteine (Yusuf and Setiarto 2022).

#### 3.1.2 Cereal‐Based Ingredients

Cereal‐based ingredients, such as oat, wheat, and rice, are also used in both PBDAs and PBMAs. These ingredients contribute carbohydrates, fiber, and micronutrients to the final products (Senarathna et al. 2024). Wheat‐derived ingredients are particularly relevant from a food safety perspective due to their gluten content and unsuitability for individuals with celiac disease or gluten intolerance. Rice bran protein has been explored as an ingredient in plant‐based foods due to its good digestibility and hypoallergenic nature, making it a potential protein source for consumers with cereal allergies (Rivero Meza et al. 2024).

#### 3.1.3 Other Plant‐Based Ingredients

Nut‐based ingredients contribute protein, unsaturated fatty acids, vitamins, and minerals to plant‐based foods but are also associated with a high allergenic potential, which must be considered in food safety and consumer protection (Matos et al. 2024).

In addition to legumes, other potential sources of protein in meat substitute mixtures include rapeseed, sunflower, quinoa, and other seed proteins, as well as hemp, potato, and corn, contributing to protein diversity and nutritional variability in PBMAs (Yusuf and Setiarto 2022).

### 3.2 Chemical Hazards in Raw Materials of PBDAs and PBMAs

Several foodborne hazards have been identified in the various cereals, legumes, nuts, and seeds used as raw materials for PBDAs and PBMAs. Some of them are already monitored and have legislative maximum levels, which may aid in decreasing the presence of these contaminants and microbes in the final products. In this publication, we will discuss mycotoxins among the chemical hazards.

Most of the chemical hazards present in the raw materials of PBDAs and PBMAs fall into two main groups: harmful metals or metalloids, and mycotoxins. In addition, plant protective substances like pesticides can be present due to actions of humans. Reports of other types of contaminants are rare, possibly partly because of limited research.

Harmful metals, such as cadmium (Cd), lead (Pb), nickel (Ni), and aluminium (Al), and metalloids like arsenic (As), are elements and therefore they are present in at least small amounts in all foodstuffs. Area of origin, species as well as cultivar of crop, and fertilization are among the factors affecting the concentrations of the elements in plant raw materials. Rice has a higher As content than most other foods, while nuts and oilseeds can have higher concentrations of other heavy metals; wheat has a higher Cd content than other main cereals, and oat contains Ni but is low in other heavy metals (Suomi, Valsta, and Tuominen 2021; Suomi, Uusitalo, et al. 2021). Seaweeds can have a high content of Cd, Pb, As, and iodine (EFSA 2023), and consumption recommendations have been given in some countries, including Finland, to avoid the possible too high iodine intake as well as the heavy metals.

The toxic metals can cause a multitude of health effects if the exposure is too high. These include damage to the central nervous system (EFSA 2010, 2008), kidney damage (EFSA 2010, 2009), possible reproductive effects (EFSA 2020), and cancer of the skin, lungs, or bladder (EFSA 2009, 2024).

Aflatoxins, particularly aflatoxin B1 (AFB1), are highly carcinogenic (EFSA 2020). Deoxynivalenol (DON) is acutely vomit‐inducing and chronically slows weight gain in young animals, as well as presents genotoxicity in vitro through oxidative stress (EFSA 2017). Fumonisins are toxic to liver and kidneys in animal tests (EFSA 2018) and cytotoxic and carcinogenic (Ekwomadu et al. 2021), while T2‐ and HT2‐toxins are immuno‐ and hematotoxic as well as acutely vomit‐inducing (EFSA Panel on Contaminants in the Food Chain (CONTAM) et al. 2017). Zearalenone (ZEN) has some estrogenic potential (EFSA 2016). Ochratoxin A (OTA) causes kidney damage in swine and is carcinogenic, although it is not certain whether the genotoxicity is direct (EFSA 2020). Citrinin is also toxic to kidneys (EFSA 2012). Some Alternaria toxins have structure with carcinogenic potential, while others belong to Cramer class II or III based on their structure (EFSA 2016).

Mycotoxins of interest for cereals, nuts, seeds and legumes are aflatoxins, ochratoxin A, the various *Fusarium* toxins including trichothecenes, fumonisins and zearalenone (ZEN) as well as emerging mycotoxins such as enniatins and beauvericin, and *Alternaria* toxins (Suomi, Valsta, and Tuominen 2021; Suomi, Uusitalo, et al. 2021). *Fusarium* mold is common in cereals also in temperate climates, while *Aspergillus flavus*, which produces aflatoxins, is found in hot and humid conditions. Many of the trichothecenes, including T2 and HT2 toxins and deoxynivalenol (DON), as well as aflatoxins and ochratoxin A (OTA), are monitored in the EU. DON is commonly found in cereals, for example, oats, and fumonisins in rice. Aflatoxins mainly contaminate nuts, rice and soy (soy flour), as well as cereals growing in hot and humid areas of the globe. The presence of *Monascus purpureus* in rice may also produce citrinin. Overall, the raw materials of PBDAs and PBMAs can contain several already controlled chemical hazards such as some of the heavy metals and mycotoxins. In addition, they can also contain compounds not yet included in the contaminant legislation, for example, aluminium, and emerging mycotoxins. These hazards may persist in the PBDA and PBMA production processes.

### 3.3 Microbiological Hazards in Raw Materials of PBDAs and PBMAs

The raw materials used in PBMAs and PBDAs present various microbiological hazards. The water activity in most ingredients is low, which generally prevents the occurrence of pathogens.

As sporeforming bacteria, *Bacillus cereus* is notable for its ability to withstand dry conditions. *B. cereus* is particularly common in rice (Ankolekar et al. 2009; Park et al. 2009), and cooked rice is known to frequently cause food poisoning outbreaks (Rahnama et al. 2023). *B. cereus* has also been found in various other grains (Daczkowska‐Kozon et al. 2009; Lesley et al. 2013). *B. cereus* causes two types of gastroenteritis: emetic form, which typically involves vomiting, and diarrheal form. The symptoms appear typically within a few hours (Ranasinghe and Fhogartaigh 2021).

*Salmonella* thrives in dry and fatty foods and often contaminates nuts. *Salmonella* has been detected in almonds, cashew nuts (Zhang et al. 2017, 2021), pistachios (Harris et al. 2016; Little et al. 2009; Zhang et al. 2021), brazil nuts (Little et al. 2010), hazelnuts (Zhang et al. 2017), walnuts (Davidson et al. 2015; Zhang et al. 2017), macadamia nuts (Zhang et al. 2017, 2021), and pecans (Brar et al. 2016). *Salmonella* is known to survive in nuts for extended periods, even over a year in cool conditions (Kimber et al. 2012; Uesugi et al. 2006). *Salmonella* is among the most common bacterial causes of gastroenteritis in Europe (EFSA and ECDC 2022, 2023, 2024). Symptoms associated with *Salmonella* infection include nausea and diarrhea (Ranasinghe and Fhogartaigh 2021). In addition to acute symptoms, *Salmonella* infection is associated with sequelae, which can pose significant risks, especially to vulnerable populations. For instance, salmonellosis has been linked to the development of reactive arthritis (Shafiee et al. 2024).

Walnuts have also been found to contain *Escherichia coli* O157:H7 (Davidson et al. 2015), and *E. coli* has been detected in walnuts, almonds, and Brazil nuts (Little et al. 2009). STEC infections typically cause bloody diarrhea and may lead to hemolytic uremic syndrome (HUS), a serious kidney disorder that can be particularly dangerous in children (Ranasinghe and Fhogartaigh 2021).

*Listeria monocytogenes* has also been found in nuts (Eglezos 2010). Both *E. coli* and *L. monocytogenes* can survive in nuts for long periods at typical storage temperatures, although not as well as *Salmonella* (Kimber et al. 2012). Nuts typically become contaminated from soil during harvest, but contamination can also occur during preprocessing or storage (Brar and Danyluk 2018). *L. monocytogenes* differs from other foodborne pathogens in that it can cause a more severe illness known as listeriosis, although it is also known to cause gastroenteritis. The severe form of listeriosis includes systemic infection and meningitis and is typically observed in individuals belonging to risk groups (WHO 2018).

Mukuna et al. (2021) detected from laboratory‐made PBDAs several bacterial species belonging to *Enterobacteriaceae* that showed antibiotic resistance. The studied raw materials included almonds, cashew nuts, and soybeans. The studied *Enterobacteriaceae* most often showed resistance to vancomycin (99% resistant isolates from all extracts), novobiocin (92%), and erythromycin (89%) (Mukuna et al. 2021).

Overall, the available evidence indicates that the raw materials used in plant‐based milk and meat alternatives are generally microbiologically safe, primarily due to their low water activity, which limits the growth of most pathogenic bacteria. However, nuts differ from other raw materials, as they more frequently harbor pathogenic microorganisms. *Salmonella*, in particular, is commonly detected in nuts and is known to persist in these products for extended periods. Other pathogens, as well as *E. coli* indicative of fecal contamination, have also been isolated from nuts. Cereals are considered microbiologically safe ingredients, but they may contain spore‐forming bacteria, most notably *B. cereus* , which can germinate under unsuitable storage conditions. Therefore, it is essential to prevent the growth of spore‐forming bacteria by maintaining dry and cool conditions during storage and by applying adequate heat treatments during the processing of PBDAs and PBMAs. These findings underscore that while most PBMA and PBDA ingredients are inherently of low risk, nuts require greater attention in safety management due to their pathogen load.

## 4 Chemical and Microbiological Hazards in Meat and Milk

EU legislation sets maximum levels for lead and cadmium in animal‐based products like meat and milk, and also for arsenic and nickel in infant formulae. In addition, animal‐based foods are controlled for a number of environmental contaminants like dioxins and PFAS compounds. Many of these contaminants have a low uptake in plants because of fat solubility or other reasons, and therefore they are not relevant chemical hazards in PBDA or PBMA.

For cadmium and lead, animal‐based products contribute significantly to the total dietary exposure. The contribution of ‘Meat and edible offal’ to the total cadmium exposure in Europe (EFSA 2012) was on average 8% but varied between 2%–11% for children and 6%–25% for adults in the EU Member States included in the assessment. The contribution of ‘Milk and dairy products’ varied between 1%–12% for children and 1%–4% for adults. Grains and other plant‐based raw products were the highest contributors to cadmium exposure.

The food category ‘Meat and meat products’ contributed at the median one fifth to the total lead exposure of children aged 1–10 years in Europe, varying between 11% and 37% for the different EU Member States (EFSA 2025). For the adult population, meat contributed 28% (14%–39%) to the total lead exposure and was the food group with the highest impact on the total exposure. The food category ‘Milk and dairy products’ contributed less, 5%–12% for children's and 3%–7% of adults' total lead exposure. For vegetarians, the main lead exposure source was grain‐based products (EFSA 2025).

For nickel and arsenic, animal‐based products are a less important source of total dietary exposure. The contributions of “Meat and meat products and Milk and dairy products” to the total dietary exposure to nickel in Europe were both mainly between 1% and 5%, except for toddlers, for whom the contribution from dairy was up to 10% in more than half of the national estimates (EFSA 2020). Most of the European monitoring data on inorganic arsenic in milk and dairy are amounts below the limit of quantification of the analysis methods, with only a few percent of numerical results (EFSA 2021). Thus, under the lower bound estimate (where < LOQ is counted as 0), milk and dairy are an insignificant (< 0.1%) source of inorganic arsenic. Likewise, the food group ‘meat and meat products’ only contributed at a maximum 3% to the total dietary exposure to inorganic arsenic under the lower bound estimate (EFSA 2021).

In Europe, mycotoxin occurrence in animal‐based foods is scarce, or the concentrations are mainly low, according to the above referenced EFSA reports. EFSA uses in their assessments national control data from EU Member States, and therefore their datasets are usually much larger than individual research projects can provide. No results above the limit of detection were found either for aflatoxins in meat (EFSA 2020) or for zearalenone in meat and meat products (EFSA 2016), and there is no relevant info on T2 or HT2 toxin in food of animal origin (EFSA 2017). DON in milk and dairy products was only measured in 4 samples, out of which one (12.5 μg/kg) was positive (EFSA 2017). OTA was reported in wild boar meat (mean 0.05 μg/kg) and in processed meat like sausages (mean 0.94 μg/kg), and Italy has considered a national maximum limit for OTA in meat because of a suspected environmental contamination of pork products (EFSA 2020).

Even though mycotoxin occurrence in the national monitoring or control samples of meat or dairy is generally scarce in Europe, literature suggests mycotoxin residues can occur in animal products (Adegbeye et al. 2020). Aflatoxin B1 is metabolized in animals to the less potent aflatoxin M1, which is occasionally found in milk as a result of aflatoxin‐contaminated feed and for which there is a maximum limit in EU legislation. Adegbeye et al. (2020) also mention mycotoxin, especially aflatoxin, contamination in eggs, meat, and offal, but feed control will keep the residues low as the source for these findings is contaminated feed. Most mycotoxins in animals are eliminated through urine, feces, or milk (Yiannikouris and Jouany 2002). Currently, mycotoxin contamination is worst in warm, wet, tropical, or subtropical climates (Adegbeye et al. 2020), but climate change can be a driving factor to increase mycotoxin contamination in crops of currently less prone areas and also the occurrence of mycotoxin residues in animal‐based foods.

A wide variety of pathogens regularly contaminate meat and milk, and products made of those. In particular, enteropathogenic bacteria such as species of the genus *Salmonella*, Shiga toxin‐producing *E. coli* , *Campylobacter* spp., some *Clostridium* spp., *L. monocytogenes*, and *Yersinia enterocolitica* frequently contaminate meat and pose possible health risks through meat consumption. Additionally, several parasites, such as *Trichinella* spp. and *Toxoplasma gondii*, can be transmitted via meat. Among viruses, hepatitis A and E viruses and norovirus can contaminate meat (Lianou et al. 2017). In Europe, *Salmonella* causes the most infections among meat‐borne pathogens, although there are significant regional differences within Europe. *C. perfringens* and *Campylobacter* have also been significant causes of meat‐borne food poisoning outbreaks in Europe in recent years. Milk causes fewer food poisoning outbreaks than meat (EFSA and ECDC 2022, 2023, 2024). Many of the pathogens are the same as those found in meat, as milk is often contaminated by pathogens originating from the environment and present in animals (Oliver et al. 2005).

The hazard profiles of plant‐based and animal‐based foods differ markedly owing to their distinct biological origins and production systems. Plant‐based raw materials generally contain a broader spectrum of chemical contaminants than animal‐derived foods. In particular, mycotoxins are frequently detected across a variety of plant commodities, whereas their presence in foods of animal origin is comparatively rare. Conversely, microbiological hazards include several organisms that are inherently associated with animal‐derived foods. For example, many parasites require an animal intermediate host to complete their life cycle and are therefore seldom transmitted via plant‐based foods. Similarly, numerous bacteria and viruses are naturally carried by animals, making animal‐based products more susceptible to contamination by these pathogens. Taken together, while plant‐based ingredients are primarily characterized by chemically driven risks, animal‐derived foods are predominantly associated with microbiological hazards, underscoring the need for rigorous control measures throughout primary production, processing, and distribution.

## 5 Plant‐Based Dairy Alternatives, Including Fermented Products & Desserts

In this chapter, the plant‐based dairy alternatives are mainly discussed as a group, rather than divided by the main ingredient, in order to facilitate comparison with each other and with animal‐based products. The nutritional factors and a selection of national recommendations are discussed briefly, and the focus is on chemical and microbiological food safety aspects.

Plant‐based dairy alternatives are made from many different types of plants. For the drinks, the most common raw ingredients are soy, almond, oat, rice and coconut, but many other alternatives also exist. The ingredients mainly fall in the following categories: legumes (soy and pea), nuts (almond, cashew, hazelnut, macadamia, coconut, tiger nut), cereal grains (oat, rice, rye, millet, quinoa), seeds (hemp, flaxseed) and roots (potato). For yoghurts, cheeses and vegan ice‐creams, the ingredients mostly belong to the groups legumes, nuts, or cereal grains. Some plants are used for these products that are not yet generally used for drinks, for example, broad bean, but the most common ingredients are the same between the PBDA types.

Fermented plant‐based dairy alternatives, such as plant‐based yoghurts and probiotic drinks, differ microbiologically from non‐fermented PBDAs due to the use of starter cultures and the associated acidification of the product matrix (Harper et al. 2022). These products are typically fermented using lactic acid bacteria (LAB), including species of *Lactiplantibacillus*, *Lacticaseibacillus*, *Leuconostoc*, *Streptococcus*, and *Bifidobacterium*, often adapted from dairy applications to plant‐based substrates. Processing raw materials into commercial plant‐based drink alternatives (PBDAs) involves multiple technological steps. Initially, the raw materials undergo pretreatment, such as dehulling, soaking, or blanching. After pretreatment, the materials are milled, and macronutrients and other soluble compounds are extracted from the resulting flour. The solid fraction is then separated from the liquid, after which additional ingredients, including stabilizers, emulsifiers, and flavorings, are incorporated. The mixture is subsequently homogenized to achieve a stable suspension and desirable texture. Typically, PBDAs undergo heat treatment to reduce microbiological contamination, after which they are packaged in carton or plastic containers (Reyes‐Jurado et al. 2021). Overall, PBDA manufacturing is a complex, multi‐stage process, and the fate of both chemical and microbiological hazards during processing remains insufficiently understood.

### 5.1 Nutritional Factors

Nutritional factors of plant‐based dairy alternatives have been studied in greater depth than from the food safety hazard point of view. In this article, the review of nutritional factors will concentrate on findings with possible health risks or benefits compared with cow's milk. Table 1 compares the main differences between PBDAs and cow's milk.

**TABLE 1:** Major differences between cow's milk and liquid plant‐based dairy alternatives (PBDA). Data are collected mainly from Antunes et al. (2022), and other sources are marked by numbers: (1) Brooker et al. (2023), (2) Clegg et al. (2021), (3) Redan et al. (2023), (4) Giugliano et al. (2023).

Factor	Difference	Significance to health
Dietary fiber	No fiber in milk, varying fiber content in PBDAs	Dietary fiber is beneficial
Sugars	Lactose not found in PBDAs. Sugar profiles of PBDAs vary with type.	Lactose intolerant can use PBDAs
Glycemic load	Almond and soy PBDAs have GL comparable to milk, oat and especially rice PBDAs have higher GL.	Important to consider for diabetics
Fats and cholesterol	Much variance between PBDAs. Coconut PBDA high in saturated fats, other PBDAs have slightly higher PUFA content than milk. Cholesterol is not present in PBDAs.	Important for cardiovascular health
Protein	Soy PBDA comparable with milk, cereal‐based PBDAs have lower protein levels and also protein digestibility slightly lower than for milk. Depending on source, PBDA protein may not contain all essential amino acids.	Possibility of too low protein intake in fragile population groups if milk is replaced by low‐protein PBDAs
Calcium: phosphorus ratio	Milk has recommended Ca:P ratio for bone health, all PBDAs have higher, especially oat and almond PBDAs. 3: Milk P content is statistically significantly higher than that of almond and coconut PBDAs, but pea‐based PBDA had more P than milk. 4: rice PBDA has higher Ca:P ratio than oat PBDA, soy PBDA has Ca:P ratio between oat and cow milk.	Unbalanced Ca:P ratio may lead to negative consequences on bone health
Vitamins	Non‐organic PBDAs often fortified, but organic PBDAs are not fortified. Fortification usually focuses on Ca, vitamins D, B2, B12 and iodine. 2: B2, B12 and iodine levels in milk significantly higher than the PBDA average	Possibility of hypovitaminosis (rickets, scurvy) or goiter with non‐fortified PBDAs. Antunes et al. (2022) consider non‐fortified PBDAs contraindicated for infants and children.
Iodine		
Other micronutrients	3: Mg highest in soy PBDAs and lowest in oat PBDAs, at least in samples from USA	

Plant‐based yoghurt alternatives have also been studied from the nutritional point of view. Boeck et al. (2021) discuss yoghurt alternatives made of pulses. They generally have high protein and lysine content as well as phenols, polyphenols, saponins, and flavonoids. Antinutritional factors such as phytates can also be present and thus decrease the bioavailability of nutrients, but they can be decreased by fermentation. Boeck et al. (2021) see pulses‐based yoghurts as a good alternative to soy, coconut, or almond yoghurts. Craig and Brothers (2021) studied the nutritional composition of yoghurt alternatives made of coconut, almond, soy, legumes, nuts or seeds, oats, or mixtures thereof. Many of the products had fiber content according to the recommendations, but the yoghurt alternatives were not fortified as frequently or to the same levels as the corresponding drinks. Compared with plant‐based dairy alternatives, plant‐based yoghurt alternatives had higher fat, sugar, protein and energy content and less Na, Ca and vitamin D.

Milk possesses certain characteristics that enhance the absorption and bioavailability of nutrients that are not found in plant‐based dairy alternatives (Rasika et al. 2021). For example, casein in milk coagulates and forms a gel that slows down metabolism, enabling more time for nutrient digestion and absorption. In addition, lactose in milk increases the bioavailability of calcium and other minerals. In plant‐based milk alternatives, the bioavailability of nutrients and minerals is lower than in milk of animal origin. For example, phytic acid in oat, soy, and cashew drinks creates insoluble complexes by binding essential minerals and trace elements, inhibiting their absorption. In addition, protein inhibitors and other protein compounds found in plant‐based milk alternatives lower glucose and protein absorption in the intestine. Strategies to overcome the low protein content and low bioavailability of vitamins and minerals in plant‐based milk alternatives have been suggested and tested. Fermentation, fortification with essential minerals and vitamins like B12, the addition of lacking enzymes, and mixing various plant‐based ingredients to increase the protein content have been used to create a product with similar nutritional value as milk.

Cheese alternatives were studied from a nutritional point of view mainly from cashew‐based products, which were high in unsaturated fatty acids like linoleic and oleic acid as well as palmitic and stearic acid (Jaeger et al. 2024). The authors assumed that microbes typical for dairy products, for example, the dominant bacterial species *Lactococcus lactis* and *Leuconostoc mesenteroides* , were inoculated into the cheese alternatives. The main fungal species in the study were *Geotrichum candidum* and *Penicillium camemberti*.

#### 5.1.1 Nutrition Recommendations

Recommendations on the use of PBDAs for infants and young children are not uniform. Antunes et al. (2022) consider the use of PBDAs contraindicated to children because particularly non‐fortified product use can lead to deficiency diseases, see details in Table 1. Brusati et al. (2023) reviewed recommendations, which agree that PBDAs should not be used as breast milk or formula replacing drink during the first year of life. Recommendations vary whether PBDAs are acceptable from age 12 months or age 2 years onwards. In contrast, the Food Standards Australia and New Zealand (Brooker et al. 2023) sees that PBDAs are not suitable as a complete milk replacement for children younger than 5 years. Finland (National Nutrition Council 2019) considers that for infants, only breast milk or formula should be given. For older children, if mainly PBDAs are used as drinks, the calcium, vitamin D, and iodine content of the products need to be checked to be sufficient. There is also a national recommendation in Finland (Finnish Food Authority 2025) that rice drinks should not be used as the main drink for children below school age (6–7 years) due to the inorganic arsenic content.

### 5.2 Food Safety Hazards Related to Plant‐Based Dairy Alternatives

This chapter discusses the occurrence of chemical and microbiological hazards in finished PBDA products.

#### 5.2.1 Chemical Hazards

A comparison of the mean amounts of harmful metals and metalloids found in PBDAs is presented in Table 2 for a selection of cases, for mycotoxins in Table 3 and information on the presence of other chemical hazards in PBDAs in Table 4.

**TABLE 2:** Concentrations of harmful metals and metalloids in plant‐based drinks. Units have been harmonized to μg/kg or μg/L. The table shows the range, from several publications, of the mean value for the hazard in PBDA type. The dataset of reference j is shown on a separate line for comparison. References: (a) Rubio et al. (2021); (b) Milani et al. (2023); (c) Redan et al. (2023); (d) Ruzik and Jakubowska (2022); (e) EFSA, (2021); (f) Astolfi et al. (2020); (g) Hartmann et al. (2023); (h) Marquès et al. (2022); (i) Karasakal (2020), (j) Schryvers (2025) (lower bound mean value of the dataset, or range if the units are different).

Hazard	PBDA drink main ingredient	Range of mean amounts	References
Al	Soy	176–3930 μg/L	a, b, f
Al	Soy	772 μg/kg	j
Al	Oat	65 μg/kg	f
Al	Oat	304 μg/kg	j
Al	Almond	695 μg/kg	j
Al	Rice	50 μg/kg	f
Al	Rice	375 μg/kg	j
Al	Nuts (hazelnut, peanut, walnut)	1080 μg/kg	j
total As	Rice	15.8–16.3 μg/kg	c, f
total As	Almond	0.8 μg/kg	j
total As	Oat	0.9 μg/kg	j
total As	Rice	12.8 μg/kg	j
total As	Soy	16 μg/kg	f
total As	Soy	1.1 μg/kg	j
total As	Grains (hemp, millet, spelt), coconut	0.7 μg/L–3 μg/kg	j
total As	Buckwheat	13 μg/L	j
iAs	Rice	2.3 μg/L–15.6 μg/kg	d, e
iAs	Rice	16.3 μg/kg	j
Cd	Soy	2–3.9 μg/L	a, b, c, f
Cd	Soy	3.0 μg/kg–5.6 μg/L	j
Cd	Oat	0.5 μg/kg	f
Cd	Oat	0.4 μg/kg	j
Cd	Almond	0.09 μg/kg	j
Cd	Oat, Almond, Pea	2 μg/kg	c
Cd	Rice	1.2 μg/kg	f
Cd	Rice	0.6 μg/kg	j
Ni	Soy	< 25.7 μg/L–201 μg/kg	b, g, f
Ni	Soy	255 μg/kg	j
Ni	Oat	127 μg/kg	f
Ni	Oat	130 μg/kg	j
Ni	Almond	36.6 μg/kg	j
Ni	Rice	30 μg/kg	f
Ni	Rice	57.5 μg/kg	j
Pb	Soy	2.2–10 μg/L	a, b, f
Pb	Soy	1.1 μg/kg–13.4 μg/L	j
Pb	Oat	0.2 μg/kg	h, j
Pb	Almond	0.9 μg/kg	j
Pb	Rice	5.7 μg/kg	f
Pb	Rice	0–7 μg/L	j
Pb	Cashew	15 μg/kg	f
Sn	Soy	0.3 μg/kg	f
Sn	Soy	3400–14,800 μg/kg	i
Sn	Almond	6600–20,700 μg/kg	i
Sn	Coconut	12,000–30,400 μg/kg	i
Cr	Soy	12.9 μg/kg	j
Cr	Oat	8.3 μg/kg	j
Cr	Rice	16.1 μg/kg	j
Cr	Almond	38 μg/kg (some high values)	j

**TABLE 3:** Concentrations of mycotoxins in oat, soy, almond/nut or rice drinks. The table shows the range, from several publications, of the mean value for the hazard in PBDA type. For aflatoxins, only the most toxic is shown. The average of positive mean values in the dataset of ref. h is shown on a separate line. References: (a) Miró‐Abella et al. (2017); (b) Torrijos et al. (2025); (c) Granados‐Chinchilla et al. (2018); (d) Hartmann et al. (2023); (e) Juan et al. (2022); (f) Milana et al. (2024); (g) Torrijos et al. (2026); (h) Schryvers (2024).

Hazard	PBDA main ingredient	Mean amount range for positives (μg/L)	Occurrence^a^	References
AFB1	Oat	0.09–0.3	Moderate (g) to high (b)	a, b, f, g
AFB1	Oat	0.25	Moderate	h
AFB1	Soy	0.01–0.35	Low (d) to high (b)	b, d
AFB1	Rice	0.02	Moderate	h
AFB1	Almond	0.01–0.1	Very high (low in c)	b, c, d
AFB1	Almond	0.01	High	h
AOH	Oat	0.3–0.7	Low (e, g) to moderate (b)	b, e, g
AOH	Soy	0.31	High	e
AOH	Almond/nuts	0.52	Moderate	e
AOH	Rice	0.29	Moderate	e
AME	Oat	0.06–0.17	High (g) to very high (e)	e, g
AME	Soy	0.09–0.14	Moderate (g) to high (e)	e, g
AME	Almond	0.11–0.16	Moderate (g) to very high (e)	e, g
AME	Rice	0.12	Very high	e
BEA	Oat	0.38–1.4	Moderate (e) to very high (b, g)	b, e, g
BEA	Oat	1.2	High	h
BEA	Soy	0.11–1.8	Moderate (e) to very high (g)	b, e, g
BEA	Soy	1.0	Moderate	h
BEA	Almond	0.05–1.6	Low (e) to very high (g)	b, e, g
BEA	Almond	0.26	Very high	h
BEA	Rice	0.15–1.7	Moderate	b, e
BEA	Rice	0.3	Moderate	h
DON	Oat	0.7–7.0	Moderate (g) to high (b, d)	b, d, g
DON	Oat	227 (3 high samples)	Moderate	h
DON	Soy	0.15–2.3	Low (d, g) to moderate (b)	b, d, g
DON	Almond	0.43–0.81	Moderate	b, g
DON	Almond, soy	< LOQ	Low	h
DON	Rice	17	Moderate	h
Enniatins^b^	Oat	0.2 to 0.4–0.7 to 3.2	Moderate (e) to very high (b, g)	b, e, g
Enniatins^b^	Oat	0.1 to 26	Moderate	h
Enniatins^b^	Soy	0.1 to 0.2–0 to 2.7	Moderate (e) to very high (b), all < LOQ in ref. g	b, e, g
Enniatins^b^	Soy	0.02 to 22	Moderate	h
Enniatins^b^	Almond	0.05 to 0.1–2.0 to 55	Low (e, g) to very high (b, g)	b, e, g
Enniatins^b^	Almond	0.07 to 10.1	Very high	h
Enniatins^b^	Rice	0.07 to 0.19–0 to 4.1	Moderate (e) to very high (b)	b, e
Enniatins^b^	Rice	0.03 to 0.82	Low	h
OTA	Oat	0.13–0.31	Moderate to high	a, b, f, g
OTA	Oat	0.15	Moderate	h
OTA	Soy	0.03–0.47	Moderate (a, b, f) to high (g)	a, b, f, g
OTA	Almond	0.14–0.30	Low (g) to high (b)	b, g
OTA	Almond	0.03	Moderate	h
OTA	Rice	0.40	Moderate	b
OTA	Rice	< LOQ	Low	h
STC	Oat	0.015	High	d
STC	Oat	0.05		h
STC	Soy	0.0003	Moderate	d
STC	Almond	0.009	High	d
STC	Rice	0.19		h
T2	Oat	0.32–0.97	Moderate	a, b, g
T2	Oat	0.74	Moderate	h
T2	Soy	0.10–0.41	Moderate (g) to high (b)	b, g
T2	Almond	0.21–2.2	Low (e) to moderate (b, g)	b, e, g
T2	Rice	0.36	Low	b
HT2	Oat	0.65–1.2	High (b) to very high (g)	b, g
HT2	Oat	1.46	Moderate	h
HT2	Soy	0.19–1.33	Low (g) to moderate (b)	b, g
HT2	Soy	4.6	Low	h
HT2	Almond	0.26–3.6	Low (e) to high (b)	b, e, g
TEN	Oat	0.25–16.6	Low (e) to high (b)	b, e
TEN	Oat	2.9		h
TEN	Soy	0.15	Very high	b
TEN	Almond	0.16	High	b
TEN	Almond	15.1		h
TEN	Rice	0.10	Very high	b
ZEA	Oat	0.28–5.7	Low (e) to moderate (b)	b, e
ZEA	Oat	4.9	Low	h
ZEA	Soy	0.20–10.8	Low (e) to high (b)	b, d, e
ZEA	Soy	1.8	Low	h
ZEA	Almond	0.11–3.1	Moderate (e) to high (b)	b, e
ZEA	Almond	0.57	Moderate	h
ZEA	Rice	0.12–15.6	Moderate (e) to high (b)	b, e
ZEA	Rice	11	Moderate	h
Fumonisins B1 and B2	Oat	0.4 (both)	Moderate	g
Fumonisins B1 and B2	Soy	0.3 (both)	Moderate	g
Fumonisins B1 and B2	Almond	0.3 (FB1), 0.4 (FB2)	Moderate	g

Abbreviations: AFB1, aflatoxin B1; AME, alternariol monomethyl ether; AOH, alternariol; BEA, beauvericin; DON, deoxynivalenol; HT2, HT2‐toxin; OTA, ochratoxin A; STC, sterigmatocystin; T2, T2‐toxin; TEN, tentoxin; ZEA, zearalenone.

^a^ Low 0%–10%, moderate 11%–49%, high 50%–89%, very high 90%–100%. Blank for ref. h indicates only one row in the database.

^b^ Enniatin A, enniatin A1, enniatin B, enniatin B1. The range “x to y” is for the individual compounds.

**TABLE 4:** Other chemical hazards reported in PBDAs. References: (a) Giugliano et al. (2023), (b) Marchart et al. (2019), (c) Yalçin et al. (2025), (d) Milana et al. (2024), (e) Butovskaya et al. (2025), (f) Mandelli et al. (2025).

Hazard	PBDA type	Concentration	Comment	References
Pesticides	Soy, rice, oat	Some residues	Only found in a few samples, none in organic PBDA	a
Pesticides	Soy, oat, almond, rice, also mixtures in b	None quantified	239 pesticide residues included in (e), 0/42 positives; 0/60 positives for glyphosate in (b)	b, e
Chlorate	Soy	12 μg/kg	3/15 positives for legume‐based samples; value for unflavoured drink, flavored soy drinks had more chlorate	b
Chlorate	Nuts	15–519 μg/kg (min‐max)	8/11 positives	b
Chlorate	Cereal‐based	34–166 μg/kg (min‐max)	8/22 positives	b
Chlorate	Rice‐almond mix	541 μg/kg	2/8 positives for mixture drinks; this was highest value of PBDAs	b
Melamine	Coconut, soy	170 and 57 μg/kg	Highest value of taken samples; no melamine found in oat, almond or hazelnut PBDA	c
Bisphenol A	Oat, Soy, Rice, Coconut, Almond	2 to 18 μg/L	Highest value in oat PBDA, lowest in rice	d
Bisphenol B	Rice	5 μg/L	None reported in other PBDAs of the publication	d
Atropine	Soy	0.4 to 1 μg/kg	Soy 3/12 positives; not found in other PBDAs; positive concentrations at least two times higher than ML for herbal‐based infusions	e
Heterocyclic aromatic amines	Soy	13.7 μg/L	Sum of 10 compounds. Commercial, sweetened and pasteurized drink had higher HAA content than homemade soy drinks.	f
Heterocyclic aromatic amines	Nuts	1.4 to ca. 5 μg/L	Sum of 10 compounds in commercial almond, cashew and peanut drinks	f

Rubio et al. (2021) measured metals in soy PBDAs sold in Spain. The samples had high aluminium (Al) content, but they estimated that for adults, at 500 mL/day consumption of soy PBMA, the exposure to harmful metals is of minimal risk assuming that the drink is the only source of the hazard. The French total diet study (Leblanc et al. 2011) reported low (upper bound 0.76 mg/kg) Al levels in milk, which is 5 times less than the Al content measured by Rubio et al. (2021) in soy PBDA. Milana et al. (2024) reported the Al content in plant‐based yoghurts ranging from 1.5 (coconut) to 9.0 (soy) mg/kg, which also indicates a possible increase in the dietary exposure for the consumers of these products. In our national risk assessment (Suomi, Valsta, and Tuominen 2021; Suomi, Uusitalo, et al. 2021; Suomi et al. 2020), the mean Al exposure of adults from other sources than milk and dairy was calculated to be ca. 200 μg/kg bw/week, or 20% of tolerable weekly intake (TWI). If the dairy in the diet was replaced by a daily 500 mL consumption of soy PBDA at the mean Al level reported by Rubio et al. (2021), the mean exposure of Finnish adults would be roughly doubled, but it would remain below TWI of Al.

Astolfi et al. (2020) compared the concentrations of heavy metals and other elements between milk and different types of PBDAs. They found significantly higher amounts of arsenic (As) in soy, rice, or hazelnut‐based PBDAs than in milk; the highest total As content was found in hazelnut drinks, 17.2 ± 8.2 μg/kg. Currently, only the arsenic content of rice drinks is controlled in EU legislation, but the results of Astolfi et al. (2020) indicate that more data should be collected on the As occurrence in nut‐based drinks to judge whether control measures would be needed on these as well. The heavy metal levels in PBDAs did not constitute a health risk in the estimation of Astolfi et al. (2020). Likewise, in the estimation of the Max Rubner Institut (Hartmann et al. 2023), the Cd and Pb content of oat, almond, and soy PBDAs was low, and the Ni content of these drinks would lead to a maximum exposure of 12% of the TDI if 500 mL were consumed by a 65 kg individual.

Higher nickel content in soy drinks was also reported by EFSA (2020), with mean lower bound (nondetects calculated as 0) content of 276 μg/kg in the monitoring data collected from EU member states. This value exceeds the concentrations reported in sources of Table 2, but the monitoring data are mainly from risk‐based sampling while the research articles likely use more random sampling. For comparison, the corresponding Ni content of cow's milk in the same EFSA report was only 15 μg/kg. Among cereals, buckwheat, millet and oat are known to take up nickel from the soil more efficiently than other cereals, although in the EFSA report their Ni content is lower than that of soybeans. The mean Ni content of soy drinks reported by EFSA was such that, assuming a 70 kg person consumes 250 g of the drink, the margin of exposure with the lowest observed adverse effect level 4.3 μg/kg bw is only 4.4. This means that the risk of eczematous flare‐up reaction (contact dermatitis) in nickel‐sensitized persons cannot be ruled out. The Ni amounts reported by Milana et al. (2024) for soy and coconut yoghurts, upper level 256 and 700 μg/kg respectively, also lead to the same conclusion on nickel‐sensitized persons.

The current maximum limits (ML) for the heavy metals set in Commission Regulation (EU) 2023/915 are, for raw milk, Pb 20 μg/kg, and for liquid infant formulas, Pb and iAs both 10 μg/kg and Cd 5 μg/kg. There is also a ML for iAs in nonalcoholic rice drinks, 30 μg/kg. The mean concentrations of Cd and Pb in the Finnish monitoring data of dairy products are much lower than the ML for liquid infant formulas. To roughly estimate the effect of replacing dairy in the diet of Finnish adults by alternatives with heavy metal concentrations equal to the ML for liquid infant formulas, we calculated that the mean exposure to Cd from the whole diet, as reported in (Suomi, Valsta, and Tuominen 2021; Suomi, Uusitalo, et al. 2021), would increase by 20% and that of Pb by 7%.

Considering the SD of the analysis, some of the cashew PBDAs reported by Astolfi et al. (2020) exceeded the ML for Pb in raw milk. Rice, hemp, soy, and hazelnut PBDAs can exceed the ML for iAs in liquid infant formulas, although the reported values are mainly within the ML for rice drinks. In addition, Rubio et al. (2021) refer to metal concentrations in Thailand soy drink studied by Nookabkaew et al. (2013), which exceeded manyfold the current MLs of Cd and Pb in liquid infant formulas. Since the values of the Thailand study are nearly two orders of magnitude higher than the other concentrations in Table 2, they are excluded as outliers from this discussion. Upper level total As concentrations in coconut drinks (1.3 μg/L) and coconut yoghurts (10.6 μg/kg) reported by Milana et al. (2024) were nearly three times higher than the concentrations they reported for corresponding rice‐based PBDAs.

The Austrian authority AGES (Marchart et al. 2019) measured the highest total As content in full‐corn rice drinks (max. 59.8 μg/kg, which would likely exceed the ML for iAs as the general estimate is that 70% of total As is inorganic). Pb was mainly found in PBDAs made of legumes like soy, although at concentrations below those allowed for liquid infant formulas, and the highest Cd content was measured in soy drinks (12.9 μg/kg).

In their recent report, EFSA (2025) reported generally low levels of Pb in PBDAs: 90% to 100% of the monitoring samples in drinks identified by raw material type were below LOQ. However, occasional high concentrations were reported for soy drinks (max 40.1 μg/kg in LB scenario) and rice drinks (max 50 μg/kg). Soy yoghurt, which only had 4 samples reported, had mean Pb concentration 78 μg/kg and maximum 120 μg/kg, although with the limited dataset it is not possible to conclude this is a general trend, especially considering the risk‐based sampling by food authorities. Our at the moment still unpublished data on soy yoghurt samples show < LOQ levels of Pb.

Gil‐Serna et al. (2020) reviewed the presence of mycotoxins in functional beverages, including PBDA. The raw products from which PBDA are made can be contaminated by several different types of mycotoxins, including those already included in the EU legislation (EU) 2023/915 as well as emerging mycotoxins. Table 3 and the following references show that these mycotoxins can survive the PBDA production process, although the occurrence and prevalence of these compounds show variation between publications.

Rehagel et al. (2022) measured five mycotoxins (aflatoxin B1, ochratoxin A, T2/HT2, sterigmatocystin and deoxynivalenol) in heat‐treated PBDA sold in Germany. The concentrations were mostly below or near limit of detection, but 23 out of 54 samples were positive for one or several mycotoxins when measured with enzyme immunoassays (EIA). The most found mycotoxin in the PBDAs was T2/HT2, which was positive in EIA in 12/14 oat, 1 soy and 8 other types of PBDA. The authors noted that PBDA matrix negatively affected EIA method and the reliability of the results.

Max Rubner Institut researchers (Hartmann et al. 2023) measured aflatoxin B1, ochratoxin A, T2 + HT2, sterigmatocystin, zearalenone, and deoxynivalenol in oat, almond, and soy PBDAs with low detection limits. OTA was not found in any of the samples, ZEA only in one soy drink, but the other mycotoxins had varying prevalence in the drinks. AFB1 was found in almost all almond drinks, but only one soy drink and not in oat‐based PBDA. Sterigmatocystin was found in all three drink types, more often in oat and almond‐based ones (two‐thirds of the samples positive) than in soy‐based ones. DON and T2 + HT2 were mainly found in oat‐based PBDA, with only 1–2 positive soy drink samples out of 12 studied. The Austrian AGES (Marchart et al. 2019), on the other hand, did not find any mycotoxins in PBDAs at concentrations above the LOD, which were between 0.05 μg/kg for the sum of aflatoxins and 100 μg/kg for DON.

Arroyo‐Manzanares et al. (2019) studied Fusarium toxins as well as emerging mycotoxins like enniatins and beauvericins in PBDAs. They reported oat‐based PBDA most (6 out of 8) samples positive for enniatins ENB, ENB1, ENA, and beauvericin BEA, and two samples also positive for enniatin ENA1. For soy PBDA, the same compounds were found with a lesser prevalence (2 out of 8 samples positive). Rice PBDA did not contain these emerging mycotoxins in measurable amounts.

Likewise, Juan et al. (2022) reported a high prevalence of mycotoxins in plant‐based drinks in Valencia, Spain. Of the 56 samples studied, 95% were positive for at least one of the mycotoxins in the 16‐compound multianalysis method. Oat (100%) and soy‐based (93%) drinks were the most contaminated, although according to the risk assessment in the paper, none of the mycotoxins except aflatoxin B2 found in almond drinks is a danger to the consumers' health in exposure amounts via drinks. Enniatins were the most common mycotoxins in the drinks analyzed in Spain, and Alternaria toxins AOH and TENT were found in oats (both) and almond (only TENT) based PBDAs. Many of the samples were contaminated by several mycotoxins, mainly emerging mycotoxins enniatins and beauvericin, as well as by zearalenone and HT2 toxin. The results indicate that the so‐called emerging mycotoxins should be monitored in PBDAs.

Torrijos et al. (2025) measured 19 monitored or emerging mycotoxins in 96 commercial PBDAs available in Italy, and later 120 plant‐based drinks and 92 PBMAs from UK (Torrijos et al. 2026). The studied toxins were aflatoxins, Alternaria toxins AOH, AME and TEN, fumonisins FB1 and FB2, toxins HT‐2 and T‐2, ochratoxin A, deoxynivalenol, zearalenone, four enniatins and beauvericin. High co‐occurrence of mycotoxins was seen in soy, oat or almond/nut‐based drinks (Torrijos et al. 2025 and 2026). Rice‐based drinks only had co‐occurrence of 6 mycotoxins, but all of the studied toxins, except HT‐2 and DON, were detected also in this drink type (Torrijos et al. 2025). Enniatins as well as Alternaria toxins AME and TEN were found in (nearly) all samples studied by Torrijos et al. (2025), and the prevalence of aflatoxin contamination was also high, with AFB1 found in 50% (rice) to 96% (almond or other nuts) of the PBDA samples and AFB2 even more frequently except in rice drinks. AFG1 and AFG2 were also found in 19% to 84% of the PBDAs studied. As aflatoxin production is most common in warm and humid climate, the production location (both of the crops and of the PBDAs) is expected to have a role in the probability of aflatoxin occurrence in PBDAs. With the current climate conditions, aflatoxins can be expected to be scarce in PBDAs produced in for example, Northern Europe.

Schryvers et al. (2025) collected a database (Schryvers 2024) on mycotoxins in plant‐based dairy and meat alternatives for their risk ranking assessment, and another database on metals in PBDAs and PBMAs (Schryvers 2025). They estimated that the main mycotoxin group of concern in PBDAs is aflatoxins (particularly in almond and oat drinks) (Schryvers et al. 2025). The collected dataset had a high percentage of PBDA samples positive for emerging mycotoxins, including enniatins and beauvericin, and Alternaria toxins.

Table 3 compares mycotoxin levels in the four most common PBDA types. Generally, emerging mycotoxins were reported with higher prevalence in the studies with the most sensitive methods, suggesting that these mycotoxins are widely present in PBDAs at low concentrations. The emerging mycotoxins are not yet currently regulated, and therefore monitoring data on them is scarce. Based on the results collected to Table 3 and discussed above, further study in the occurrence of these compounds in PBDAs, including the assessment of whether plant species and origin are correlated with the concentrations, would be valuable.

A chemical hazard associated with legumes, particularly soybeans, is the presence of phytoestrogens, including the isoflavone compounds daidzein, genistein, and glycitein (Banach et al. 2022). These compounds structurally resemble endogenous human estrogens and therefore exhibit estrogenic activity in vitro. Phytoestrogens have been discussed in the context of endocrine‐related effects, including potential impacts on pubertal development, hormone‐dependent cancers, reproductive development, and thyroid function. However, according to the Nordic Council of Ministers (2020), estimated exposure to genistein from a diet substantially based on soy substitutes is not considered a health concern for pregnant women or unborn children. In contrast, a potential health concern was identified for children consuming a substantially soy‐substituted diet, highlighting the importance of considering age‐specific exposure and dietary patterns when evaluating the safety of soy‐based products. EFSA's assessment of isoflavones has primarily focused on specific population groups and sources (e.g., supplements in peri‐ and post‐menopausal women). Therefore, these conclusions cannot be directly extrapolated to all consumers, indicating that further research is needed to clarify potential risks across different age groups and dietary patterns.

Of the other chemical hazards presented in Table 4, three are a possible risk for consumers assuming a daily 200 mL consumption: the reported concentrations for bisphenol A, atropine and heterocyclic amines are such that the TDI of bisphenol A determined by EFSA can be exceeded by orders of magnitude, and the acute reference dose for atropine determined by EFSA can be exceeded with the highest concentrations. Mandelli et al. (2025) concluded on heterocyclic amines that the lifetime cancer risk in some age groups is intolerable already with one daily serving of soy‐ or peanut‐based commercial PBDA. The information on these three hazards occurring in PBDAs is only based on one publication each. Therefore, data are limited, and more research on the occurrence would be urgently needed to ascertain if control measures would be required or if the preliminary findings represent unusually high concentrations.

#### 5.2.2 Microbiological Hazards

Most PBDAs available on the market are heat‐treated using either ultra‐high temperature (UHT) or extended shelf‐life (ESL) processing (Giugliano et al. 2023). Consequently, most pathogens present in the raw materials are destroyed during production. Only spore‐forming bacteria may survive the heat treatment (Doll et al. 2017). UHT processing, however, should destroy even most spore‐forming pathogens (Scheldeman et al. 2006). Cross‐contamination from raw materials, equipment, or personnel to the finished product is also possible if there are deficiencies in the processing (Doll et al. 2017).

There has been limited research on the PBDA products currently available on the market. Only a few studies have tested for the presence of pathogens in PBDAs (Table 5).

**TABLE 5:** Pathogens detected in PBDAs.

Product	Micro‐organisms	Number of samples	Positives	References
Almond‐based milk alternatives	*Bacillus* spp., *Clostridium* spp., *L. monocytogenes*	12	0	Hartmann et al. (2023)
Almond‐based milk alternative	*B. cereus*	10	0	Butovskaya et al. (2025)
Oat‐based milk alternative	*L. monocytogenes* , *Salmonella*, *B. cereus*	10	1 ( *B. cereus* )	Giugliano et al. (2023)
Oat‐based milk alternative	*B. cereus*	10	0	Butovskaya et al. (2025)
Oat‐based milk alternatives	*Bacillus* spp., *Clostridium* spp., *L. monocytogenes*	12	0	Hartmann et al. (2023)
PBDAs^a^	*B. cereus*	26	3 (1 sunflower‐rice, 1 soy, 1 oat)	Kain et al. (2024)
PBDAs^b^	*B. cereus, L. monocytogenes*	138	0	Willis et al. (2024)
Rice‐based milk alternative	*L. monocytogenes* , *Salmonella*, *B. cereus*	17	0	Giugliano et al. (2023)
Rice‐based milk alternative	*B. cereus*	10	0	Butovskaya et al. (2025)
Soy‐based milk alternatives	*Bacillus* spp., *Clostridium* spp., *L. monocytogenes*	12	0	Hartmann et al. (2023)
Soy‐based milk alternative	*L. monocytogenes* , *Salmonella*, *B. cereus*	33	0	Giugliano et al. (2023)
Soy‐based milk alternative	*B. cereus*	12	3	Butovskaya et al. (2025)

^a^ 17 different ingredients or combinations.

^b^ Ingredients not specified.

Kyrylenko et al. (2023) studied the microbiological quality of raw materials used in plant‐based milk alternatives. Most of the microbes found in the tested raw materials were spores, which can cause quality issues in heat‐treated products. Among the tested bacteria, pathogens were also detected. *B. cereus* group bacteria were isolated from pea and oat flours, with 9% of *B. cereus* isolates containing the gene necessary for cereulide production.

Gützkow et al. (2024) studied the microbiological quality of ready‐to‐drink oat, soy, and almond‐based milk alternatives. Only one of the 36 samples tested, an oat drink, showed microbiological contamination with aerobic mesophilic bacteria. No pathogens were detected in any of the samples.

In the study by Giugliano et al. (2023), a few samples of soy, oat, and rice drinks showed growth of yeasts and molds, and a few samples showed growth of total aerobic mesophilic bacteria. However, the amounts were small, except for one soy drink, which had 6700 cfu/mL of total aerobic mesophilic bacteria. One *B. cereus* was detected in oat drinks (*n* = 10), but it was unable to produce cereulide.

In the study by Kain et al. (2024), only small concentrations of total microbial counts were found in PBDAs. Three samples contained *B. cereus* , which were classified as potentially hazardous because they could produce enterotoxin.

Butovskaya et al. (2025) studied the presence of *B. cereus* in soy, oat, almond, and rice drinks. Three positive samples were found in soy drinks (*n* = 12), but none could produce cereulide.

Max Rubner‐Institut (Hartmann et al. 2023) measured pathogenic bacteria in oat, almond and soy PBDAs. Pathogenic microbes were not detected in the samples and the total microbe content was low. Only one sample was positive for aerobic mesophilic bacteria, which was postulated to be due to post‐heat treatment contamination, and no other positives were found in repeat samples of the same batch.

To our knowledge, only a limited body of research has addressed the microbiological safety of fermented PBDAs. Nevertheless, available evidence suggests that fermentation of soy‐based yoghurt can effectively inhibit the growth of foodborne pathogens, provided that the fermentation process is adequately controlled (El Gawad et al. 2014; Mishra et al. 2019). In contrast, in nut‐based fermented cheeses, at least *Salmonella* has been shown to be capable of surviving (Louvau et al. 2024).

PBDAs have been linked to several food poisoning outbreaks. Multiple PBDA products were suspected to have caused a *L. monocytogenes* outbreak in Canada in 2024. This outbreak resulted in 20 illnesses, with 15 hospitalizations and 3 deaths. *L. monocytogenes* was found in the production environment of a beverage manufacturing plant (Public Health Agency of Canada 2024).

Plant‐based cheese alternatives have caused *Salmonella* and *L. monocytogenes* outbreaks. The *Salmonella* outbreak was caused by a cashew‐based brie‐like product. The outbreak was associated with *Salmonella Typhimurium* , Chester, Duisburg, and Urbana. This outbreak resulted in 20 illnesses, with 5 hospitalizations (FDA 2021). The *L. monocytogenes* outbreak was linked to a vegan cheese product. This outbreak resulted in 7 illnesses, all of whom belonged to high‐risk groups, for example, pregnant women (Leclercq et al. 2024). In 2022, an oat drink was identified to cause *B. cereus* outbreak in Europe (RASFF, Sweden, 29 affected) and caused large product recalls.

Although pathogens are rarely detected in products due to heat treatment, when they do occur, they can pose a health risk. According to studies (Bartula et al. 2023; Kain et al. 2024), *L. monocytogenes* , *S. enterica* , and *B. cereus* grow well in PBDAs. PBDAs provide a good growth medium for bacteria due to their high a_w_, carbohydrates as substrates, and sufficient nutrients for bacterial growth (Bartula et al. 2023). *L. monocytogenes* and *S. enterica* are destroyed by heat treatment but can enter PBDA through post‐processing contamination (Lindsay et al. 2021). *B. cereus* , on the other hand, may survive heat treatment and is occasionally found in plant‐based milks (Butovskaya et al. 2025; Giugliano et al. 2023). The long shelf life of PBDAs at room temperature may pose a health risk, as pathogens have time to grow. According to Bartula et al. (2023), pathogen growth did not always cause noticeable changes in the sensory properties of PBDAs, or the changes were so small that they might not be detected.

## 6 Plant‐Based Meat Alternatives (PBMA)

A meat alternative (meat analog) refers to a product in which the main protein source has been replaced with something other than meat. Different types of protein, such as soy protein, wheat gluten, bean flour, and nuts, are used as alternatives to meat, providing a comparable taste and texture. Currently, there are meat alternatives on the market where meat protein has been replaced with a plant‐based protein source such as soy or legumes, mushroom protein (e.g., Pekilo), or single‐cell proteins.

Using vegetable protein as a meat substitute is not a new concept. For example, tofu has been incorporated in the diet of Asian cultures for many centuries, dating back as early as 965 A.D. (Thavamani et al. 2020). The term meat analog was introduced in the 1960s (Sadler 2004). In this case, soy protein was used in meat substitutes such as tofu and tempeh, which are traditional meat alternatives produced by fermentation. Dry textured vegetable proteins, like extruded soy protein concentrates and wheat glutens, appeared in stores at the end of the 20th century. The first generation of meat substitutes, such as legume, soy, and grain‐based products, were not suitable for all diets due to, for example, a strong aftertaste and allergies.

In the 21st century, as health and sustainability themes grew, meat substitutes also started to appear in the food choices of ordinary consumers, and more investments have been made in the product development of meat alternatives. During the last decade, modern food production technologies have enabled the development of new meat substitutes that mimic meat's structure, taste, appearance, and functionality. The latest products are plant‐based “meat” products (e.g., Beyond Meat) and laboratory‐grown meat. The arrival of edible insects on the plates of ordinary consumers has also been predicted, but they have not yet gained a large market share in Europe. This literature review focuses on plant‐based meat alternatives (PBMAs).

### 6.1 Nutritional Factors of Meat Alternatives

If meat is replaced in the diet completely with meat alternatives, they have to provide the same nutritional value. Plant‐based meat alternatives generally have lower protein content compared to conventional meat. However, it has been established that a meat alternative with a protein content of up to 30% with a low fat/lipid level can be an excellent alternative to meat from a nutritional perspective (Bakhsh et al. 2021). Plant‐based meat alternatives lack essential nutrients like iron and vitamin B12, which must be supplemented. Plant‐based meat alternatives are a good source of fiber and phytochemicals.

Changes in nutritional and health aspects of the diet when replacing meat with meat alternatives have been studied in the UK (Farsi et al. 2022). In the study, three models were tested. In the first model, 25%, 50%, 75% or 100% of the current meat intake was replaced with a weighted mean of meat alternatives in the UK market. In the second model, different ingredient categories of meat alternatives were compared. In the comparison of vegetables, mycoprotein, a combination of bean and pea, tofu, nut, and soya were selected. In the third model, the impact of fortification of meat alternatives was studied by comparing fortified versus unfortified meat alternatives. Models indicated significant shifts in nutrients. Overall, carbohydrate, fiber, sugars, and Na increased, whereas reductions were found for protein, total and saturated fat, Fe, and B12. The study indicated significant increases in fiber intake and significant decreases in both total fat and saturated fat intake when people switched from meat to meat alternatives. The greatest decrease in fat intake was with mycoprotein (−6.12 total g/d) and nut‐based meat alternatives (−4.8 g/d saturated fat). Especially high in fiber was the nut‐based meat alternative diet, where the intake of fiber increased by +10.23 g/d. The downsides were notable reductions in total protein (nut‐based −19.8 g/d) and vitamin B12, and projected increases in the intake of Na (soya‐based [+496 mg/d] and of sugars [nut‐based +7.44 g/d]). Consumers should select meat alternatives that are low in sodium and sugar, but high in fiber, protein, and micronutrients to maintain good nutrition while reducing meat intake.

Many plant‐based meat alternatives and dairy alternative products are considered ultra‐processed foods. There has been concern about the potential negative impacts of these foods on human nutrition and health because these products often include numerous ingredients, including colors, flavors, thickeners, gelling agents, binders, structuring agents, and emulsifiers, and are manufactured using harsh processing operations (Alcorta et al. 2021). Usually, plant‐based meat alternatives are fortified with vitamins and minerals to ensure the required nutritional intake. The high number of additives in meat alternatives can be attributed to the effort to replicate the nutritional content, flavor, and appearance of meat, making these products more appealing to consumers (McClements 2023). There are various meat alternative products available on the market, each differing in terms of nutritional content and health impact. For example, tofu and tempeh are traditional plant‐based products with higher protein content that are not considered ultra‐processed foods.

In summary, plant‐based meat alternatives offer potential nutritional benefits such as increased fiber and reduced saturated fat intake, but generally have lower protein and may lack key micronutrients like iron and vitamin B12, necessitating careful selection and possible fortification. While these products can support dietary shifts away from animal meat, attention to sodium and sugar content, as well as the implications of ultra‐processing and additive use, is essential to ensure overall nutritional adequacy and health.

### 6.2 Food Safety Hazards Related to Plant‐Based Meat Alternatives

#### 6.2.1 Chemical Hazards

Research about the chemical risks of plant toxins and other chemical hazards associated with the consumption of plant‐based meat alternatives (PBMAs) is still limited. Based on earlier studies, the highest chemical risks are those caused by mycotoxins (Schryvers et al. 2025). Aflatoxins and Alternaria toxins are the main concerns in meat alternatives. Soy‐based meat alternatives had the highest risk for mycotoxins like aflatoxin B1 (AFB1) and alternariol (AOH). Table 6 shows examples of mycotoxin concentrations in PBMAs.

**TABLE 6:** Occurrence and concentrations of mycotoxins in some PBMAs. For ref. b, the mean of positive concentrations in the database is given. References: (a) Torrijos et al. (2026), (b) Schryvers (2025).

Mycotoxin group	Occurrence in ref. a	Mean concentration in positives	PBMA type with highest contamination risk
Aflatoxins	High	0.6–0.8 μg/kg (a), 6.5 μg/kg (b)	Highest prevalence and/or highest concentrations in legume‐based
Alternaria toxins	High	AME 0.4–0.6 μg/kg (a), 156 μg/kg (b) AOH 53 μg/kg (b)	Lowest concentration in cereal‐based, highest in legume‐based. Only present in legume‐based in (b)
DON	Low	< LOD to 28.7 μg/kg (a), 368 μg/kg (b)	Especially legume‐based, only one positive sample in (b)
T2 + HT2	Low to very high	T2 1.4–1.7 μg/kg, HT2 2.1–2.7 μg/kg (a); T2 3.4 μg/kg (b)	Cereal‐based highest HT2, legume‐based lowest T2 prevalence (a). T2 only present in one legume‐based sample in (b)
Fumonisins	High	35 μg/kg (b)	Highest in legume‐based (b)
ZEA	High	1.0–1.1 μg/kg (a)	Especially legume+cereal‐based
OTA	High	1.0–1.2 μg/kg (a), 2.5 μg/kg (b)	Especially legume+cereal‐based
Beauvericin	Very high	1.1 μg/kg (a)	Similar concentrations in all types
Enniatins	Very high for ENNA, ENNA1	ENNA 0.9–1.1 μg/kg (a), 324 μg/kg (b)	Especially legume+cereal‐based (a), only present in legume‐based in (b)

Substituting meat with meat alternatives also increased dietary exposure to pesticide residues (Kesse‐Guyot et al. 2023). This applied only when conventional pesticides were used, and an organically grown plant‐based diet reduced exposure to pesticide residues (Baudry et al. 2021). The alkaloid content in plant‐based foods has been studied extensively in soy‐based products and, to a lesser extent, in wheat and wheat‐based foods. However, research on the alkaloid content of other legumes remains limited (Augustin Mihalache et al. 2022). From soy‐based products, alkaloids β‐carboline norharman and β‐carboline harman were detected.

There are also chemical hazards linked to the processing and cooking of the PBMAs, as with the corresponding meat products. Acrylamide was found in commercially available plant‐based meat alternatives during domestic cooking (Abdullajeva et al. 2025). The highest concentration of acrylamide was observed in wheat‐based meat, increasing from 65.7 ± 6.6 μg/kg before heat treatment to 119 ± 12 μg/kg following heat treatment. The lowest concentrations were detected in the minced vegetable base (ranging from 2.98 ± 0.30 μg/kg to 33.6 ± 3.4 μg/kg). The study indicated that the use of additives and processing techniques has a strong influence on acrylamide formation in plant‐based meat alternatives. Other chemical hazards that can form during the processing of the PBMAs are N‐nitrosamine and heterocyclic aromatic amino acids (Gräfenhahn and Beyrer 2024).

EFSA (2020) reported a high (7350 μg/kg) nickel content in one sample of textured soy protein, and the Ni content in tofu (mean 345 μg/kg) is also much higher than that of beef (mean 95 μg/kg), pork (mean 75 μg/kg), or poultry (mean 81 μg/kg). As discussed for PBDAs, the high Ni content in these soy‐based foods means that the risk of contact dermatitis flare‐up for nickel‐sensitized persons cannot be ruled out, although the matrix is likely to make the intake of Ni into the body slower than from drink.

EFSA (2025) reported higher amounts of Pb (mean 21.6 and max 275 μg/kg) in soybean‐based PBMAs than in other PBMA types, which also included tofu (mean 6.8 and max 120 μg/kg) and a small number of samples on fermented soybean‐based PBMAs. The PBMAs not identified by their plant origin had Pb concentrations comparable with tofu. As discussed earlier for PBDAs, the results of EFSA are based on national monitoring data collected from EU member states, and as these are often sampled risk‐based, the average concentrations can be a little higher than with random sampling.

Milana et al. (2024) listed in their review mycotoxins and processing contaminants as the main chemical hazards in alternative protein sources. Likewise, according to Gräfenhahn and Beyrer (2024), the highest hazard index for food safety components in PBMAs, based on likelihood and impact, was estimated for allergens, followed by processing contaminants (particularly acrylamide) and mycotoxins. The hazard index for heavy metals in PBMAs was low due to estimated low impact.

In addition to these, Norwegian VKM (VKM 2023) listed erucic acid, perfluorinated and polyfluorinated alkyl substances (PFAS), and potato glycoalkaloids like solanine as contaminants of which plant‐based meat and dairy analogues are known or expected to be an important source. Erucic acid was mentioned as detected in PBDAs and PBMAs, but no information on the concentrations was given. If one only focuses on the sum of the four most toxicologically relevant PFAS compounds (PFOA, PFOS, PFNA, and PFHxS), according to EFSA (2020), the contribution of PBDAs to the total dietary exposure of adults was < 1% and the contribution of dairy was as low. Meat contributed slightly more to the total dietary exposure of the sum of the four PFAS compounds, but not as much as other food groups like fish. PFAS comprise a huge number of compounds with different properties and potential for plant uptake. Therefore, charting their occurrence also in PBDAs and PBMAs is useful, although the preliminary information based on the EFSA assessment and consumption data collected in the previous decade does not yet identify these foods as an important source of the total exposure to the most toxic compounds in this group. Solanine, a glycoalkaloid produced by plants of the nightshade family such as tomatoes and potatoes, would likely mainly be relevant in PBDAs and PBMAs containing significant presence of these plants. It is presented in the VKM report as a possible contaminant due to its presence in one of the possible raw materials of PBDAs and PBMAs, not as one already detected in the final products. More data are needed to confirm the preliminary information of the VKM publication.

The list of VKM (VKM 2023) also comprised many of the hazards discussed elsewhere in this review: metals and metalloids (aluminium, inorganic arsenic, cadmium, lead), perchlorate, process contaminants (acrylamide, glycidol, 2‐MCPD, and 3‐MCPD), as well as mycotoxins (aflatoxins, alternariol methyl ether, ochratoxin A, patulin, T‐2 and HT‐2 toxins, and zearalenone).

The VKM report (VKM 2023) also compared the allowed food additives in PBMA and their animal‐derived counterparts. Although the food additives overlap, PBMAs can contain some food additives that are not allowed in animal meat products, for example, colors E141, E162, E172, preservatives E200 and E202, E211 and E270, antioxidants E322 and E333, several emulsifiers, stabilizers, thickeners and anticaking agents like E410 and E525, sweeteners E950, E955 and E965 as well as other food additives. On the other hand, some food additives used in meat products are not allowed in PBMA, in particular nitrites and glutamate E621. The lack of nitrites in PBMA has an effect on the hygiene measures required from these products, as discussed more under the risk management chapter.

Plant‐based meat alternatives (PBMAs) have been reported to present a range of chemical food safety hazards, including exposure to mycotoxins, which have been most frequently documented in soy‐based products. Substitution of meat with PBMAs may influence dietary exposure to pesticide residues and certain naturally occurring alkaloids, while processing steps can contribute to the formation of contaminants such as acrylamide, N‐nitrosamines, and heterocyclic aromatic amines. In addition, several studies have reported higher concentrations of nickel and, in some cases, lead in soy‐based PBMAs compared to animal‐derived meat products, which may be relevant for sensitive population groups. Other chemical hazards identified in specific products include erucic acid, PFAS, as well as various metals and metalloids. Furthermore, PBMAs may contain food additives that are not permitted in comparable animal‐based products, potentially influencing both chemical risk profiles and hygiene management practices. Taken together, the available literature suggests that mycotoxins and processing‐related contaminants represent key chemical hazards in PBMAs, whereas the relevance of other compounds appears to be product‐ and ingredient‐specific.

#### 6.2.2 Microbiological Hazards

The raw materials utilized in plant‐based meat alternatives (PBMAs) and plant‐based dairy alternatives (PBDAs) are comparable, resulting in similar microbiological hazards associated with these ingredients. However, the specific processing techniques differ from milk alternatives, and those also affect the microbial quality and hazards. In addition, processing steps like extrusion or cooking and using different raw materials like soy, wheat, or pea proteins affect spoilage and the occurrence of pathogenic microorganisms in PBMA, compared to meat (Barmettler et al. 2025). In conventional meat, the initial microbial load can be higher due to contamination during slaughter and processing. Both PBMA and conventional meat can be contaminated during processing, with the type of spoilage and microbial community differing between them.

PBMAs are produced using raw materials, additives, and structure‐forming processes like extrusion. Heat processing is essential for achieving the desired texture and ensuring microbiological safety. Contamination risks come from spore‐forming bacteria like *Bacillus* and *Clostridium* group that survive the heating step and from re‐contamination after production or during cooking and storage (Kabisch et al. 2024).

The microbiological quality of 100 PBMA products of a broad range of products was studied in Switzerland (Barmettler et al. 2025). The total viable counts (TVCs) of the products were between < 2 log and 7 log CFU/g, with a median of 5.97 log CFU/g. *Listeria* was found in 7 of the 100 (7%) of the tested PBMAs, but none of the isolates were identified as *L. monocytogenes* . *B. cereus* group members with the ability to produce enterotoxins were detected in 5 out of 100 (5%) products. *Enterobacteriaceae* were detected in 6 out of 100 (6%) samples, with a total bacterial count of 2 log to 3 log CFU/g. *Staphylococcus aureus* was detected in 2 out of 100 (2%) samples.

Willis et al. (2024) studied the microbiological quality of vegan alternatives to dairy and meat products in England during 2022. Of the 937 samples tested, 92% were of a satisfactory microbiological quality, 3% were borderline, and 5% were unsatisfactory. Those interpreted as unsatisfactory were due to elevated counts of *Enterobacteriaceae* and *E. coli* , indicating poor hygiene during preparation. Pathogenic species *L. monocytogenes* were present in five samples of tofu; other *Listeria* species were detected in small amounts (< 20 CFU/g) from two burgers and two ‘vegan chicken’ products.

In Finland, an outbreak of listeriosis in dialysis patients exposed to raw, chilled, or ready‐to‐eat plant‐based products containing fava beans was identified in 2019 (Otte Im Kampe et al. 2024). The source of the contaminant was traced back to the manufacturing company of the fava bean product because *L. monocytogenes* was detected in the product and also in the surface samples taken from the processing environment.

Tóth et al. (2021) studied the microbial quality of PBMA raw materials and products after cooking and storage at different temperatures. Of the tested raw materials (wheat glucan, soy protein, pea protein), only wheat gluten powder showed medium contamination with a microbial load of 3.32 log10 CFU/g. *Enterobacteriaceae* were detected in two unrefrigerated PBMA samples, and yeasts were also observed in one sample of unrefrigerated vegan cabbage casserole. The pH of PBMA products was closer to 7, and the high water content and water activity of the raw material promote bacterial proliferation. Tóth et al. (2021) concluded that meals made with meat analogues pose a slightly higher food safety risk than those with real meat, necessitating greater preparation, processing, and storage attention.

The microbial quality of vegan ground beef products made from soy, pea, or oats was tested in Germany (Kabisch et al. 2024). The total bacterial counts at the end of the best‐before date varied greatly from below 1.0 log10 CFU/g to 8.31 log10 CFU/g, while the median count was 3.89 log10 CFU/g. *B. cereus* and various *Clostridium* species were detected from PBMA products, indicating that spore‐formers may have survived the food processing and therefore could pose a safety concern during storage. In addition, in January 2023, there was a recall of vegan mince in Germany in which *Salmonella* was found (Rügenwalder 2024; Barmettler et al. 2025).

The prevalence of *Clostridium botulinum* in vegan sausages was analyzed from a total of 74 samples of frozen (8) or chilled (66) packaged vegetarian sausages from seven producers in Finland and Germany (Pernu et al. 2020). A high overall prevalence of 32% for *C. botulinum* was detected in the vacuum‐backed vegan sausages, with cell counts ranging from 20 to 1200 *C. botulinum* cells/kg. The high prevalence of *C. botulinum* in vegetarian sausages indicates that these products may pose a risk for botulism. Mild heat treatments allow the survival of *C. botulinum* spores, while extended shelf life may facilitate spore germination, growth, and toxin production.

Plant‐based meat alternatives (PBMAs) have been reported to exhibit microbiological hazards comparable to those observed in plant‐based dairy alternatives (PBDAs), with risk profiles influenced by raw material composition, processing conditions, and post‐production handling. Previous studies have documented the presence of spore‐forming bacteria, including species of Bacillus and Clostridium, as well as occasional contamination with *Listeria monocytogenes* , Enterobacteriaceae, *Staphylococcus aureus* , and Salmonella. Reported microbial loads and pathogen prevalence vary between product categories and geographical regions, reflecting differences in processing practices, hygiene, and storage conditions. In particular, the documented survival of spore‐forming bacteria in vacuum‐packed or mildly heat‐treated PBMAs highlights the importance of adequate process control and storage conditions when assessing microbiological food safety.

## 7 Safety Challenges Across Processing Stages of PBDAs and PBMAs

Processing plays a central role in shaping the food safety of PBDAs and PBMAs. Because these products rely on diverse raw materials and undergo a wide range of technological treatments, processing can either mitigate or amplify specific chemical and microbiological hazards, and certain hazards may persist beyond processing. This chapter summarizes key microbiological and chemical risks in different processing steps used in the commercial production of PBDAs and PBMAs and evaluates their effectiveness in controlling known hazards, highlighting areas where risks persist or require additional attention.

As discussed earlier, most PBDAs and PBMAs are not currently subject to monitoring for contaminant maximum levels like the corresponding animal‐based products are. Thus, legally required risk management of these products largely relies on quality control of the raw materials.

### 7.1 Plant‐Based Dairy Alternatives

**FIGURE 1:**
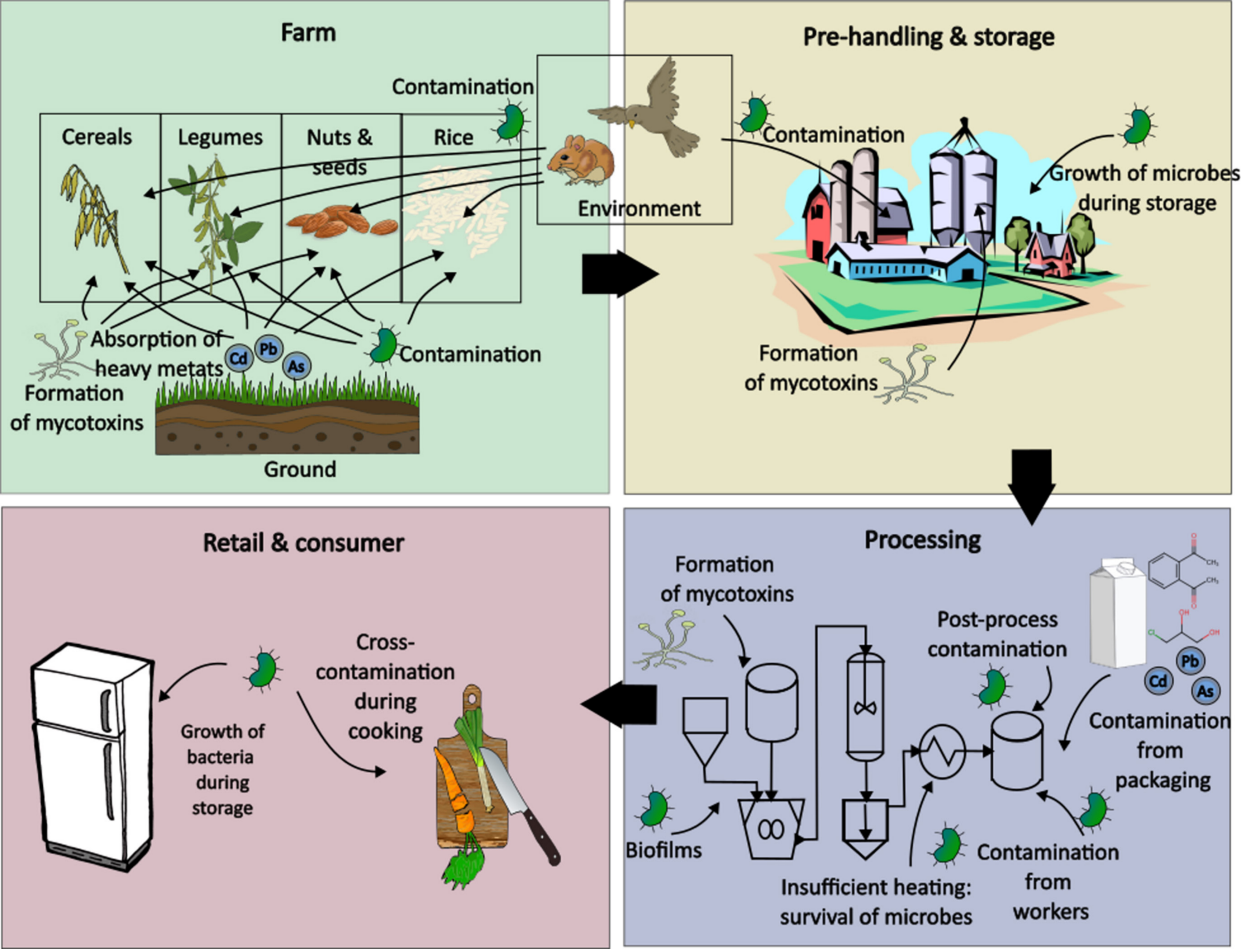
Potential contamination points and associated hazards along the production chain of plant‐based milk alternatives.

Contamination with microbiological or chemical hazards can occur at any point along the food production chain (Figure 1); however, the risk is particularly pronounced during the early stages. Heavy metals such as cadmium, lead, and arsenic can be absorbed by plants directly from the soil during growth, with the amounts dependent on plant species and cultivar as well as growing conditions, naturally occurring levels in soil as well as additional burden from fertilization, pollution, etc.

Soil also serves as a natural reservoir for various pathogens, and crops that come into contact with it may become contaminated with organisms such as *L. monocytogenes* , *B. cereus* , *Salmonella* spp., or *E. coli* . This risk is especially relevant for nuts, which frequently encounter soil at multiple stages of production. In addition to direct soil contact, contamination during primary production may also occur via environmental vectors such as surface water or pests. Under favorable environmental conditions, fungal growth may be initiated, potentially leading to the production of mycotoxins (Brar and Danyluk 2018).

To mitigate these risks, good agricultural practices, for example, use of clean water sources and fertilizers, and maintaining the separation between animal husbandry and crop production, are essential (FAO 2004).

During storage and processing, microbial proliferation may continue if conditions such as temperature and humidity are conducive to growth. Fungi, in particular, may continue to produce mycotoxins during storage if environmental controls are inadequate. Furthermore, contamination from external sources, including pests, remains a concern throughout the storage phase (Brar and Danyluk 2018). The processing stage introduces additional contamination points, including the formation of biofilms and mycotoxins, insufficient thermal treatment, and post‐process contamination from equipment or personnel (Faille et al. 2018; Le Gentil et al. 2010).

The microbiological quality of PBDAs is controlled through appropriate temperature management and by preventing contamination, for example, by adhering to hygienic working practices. Food production facilities manufacturing PBDAs are required to implement a Hazard Analysis and Critical Control Points (HACCP) plan, which enables the identification and control of hazards that are critical to food safety ((EC) No 852/2004).

Fermentation represents a distinct processing step that modifies the microbiological hazard profile of PBDAs compared with non‐fermented products. The safety of fermented PBDAs relies on multiple interacting factors, including starter culture selection, fermentation kinetics, pH dynamics, and storage conditions. Fermentation has been shown to improve product stability by inhibiting the growth of spoilage microorganisms and foodborne pathogens through acidification and the production of antimicrobial metabolites (Zapaśnik et al. 2022; Mokoena et al. 2021). Several studies have shown that effective LAB fermentation in plant‐based matrices can reduce pH to below 4.5, thereby inhibiting pathogens such as *L. monocytogenes* , *Salmonella* spp., and *E. coli* (Ziarno et al. 2023; Tabanelli et al. 2024). However, insufficient acidification, inappropriate starter culture performance, post‐fermentation contamination, or inadequate temperature control during storage may allow the survival or growth of acid‐tolerant pathogens also in fermented PBDAs (Zapaśnik et al. 2022).

Packaging materials may act as sources of contaminants, potentially introducing unwanted substances into food products. However, food packaging within the European Union must comply with legally mandated safety requirements ((EC) No 1935/2004). Currently, there are no specific legislative provisions concerning PBDAs in relation to food packaging.

The consumer represents the final line of defense in ensuring food safety. Improper storage conditions may facilitate the growth of bacteria or molds, while inadequate food handling practices can lead to cross‐contamination. Unlike earlier stages of the food supply chain, consumer behavior cannot be regulated through legislation. Nevertheless, consumers can be educated on the importance of temperature control and safe food handling practices in the domestic kitchen environment (Jevšnik et al. 2022).

### 7.2 Plant‐Based Meat Alternatives

**FIGURE 2:**
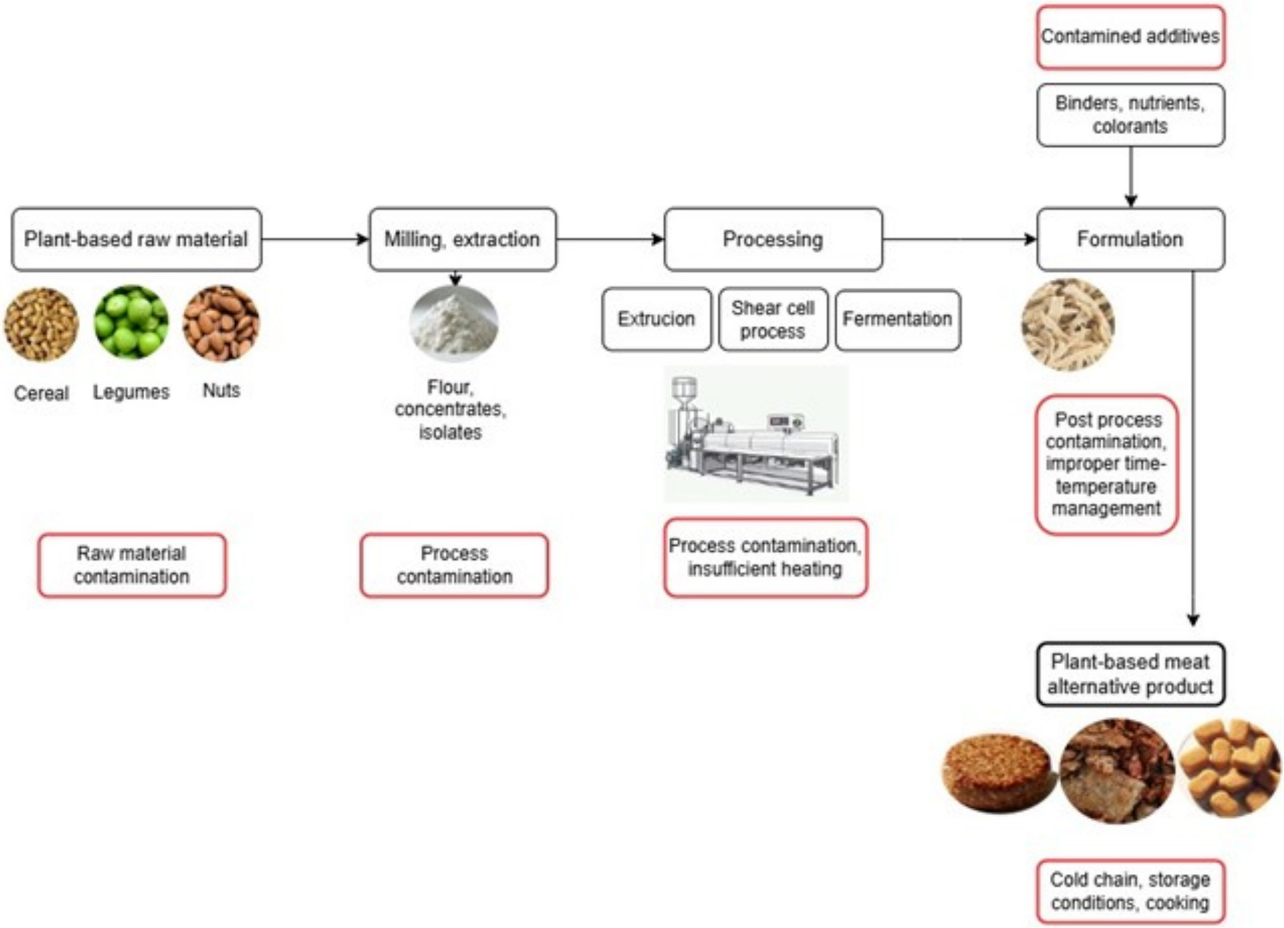
Flow chart of the production process of plant‐based meat alternatives and microbiological risks related to the manufacturing process (in red).

Plant‐based raw materials such as cereals, legumes, and nuts can be utilized either whole or as protein concentrate ingredients in the production of plant‐based meat alternatives (Figure 2) (Banach et al. 2022). These raw materials may have been contaminated in the field and carry pathogenic microbes from soil and animal feces that can be transferred to the final PBMA product. Raw materials typically go through a heating or drying step in the protein extraction step (Banach et al. 2022; Thakur et al. 2024). Heat treatment in the processing step eliminates most of the bacteria, excluding thermal‐resistant spore‐forming bacteria (*Bacillus* and *Clostridium* group) that can remain viable (Wild et al. 2014) (Table 7). With an increase in the number of ingredients, the potential sources of microorganisms in the product also increase.

**TABLE 7:** Impact of processing on food safety hazards in PBDAs and PBMAs.

Hazard category	Processing step to control hazard	Hazard persisting or exacerbated after processing
Vegetative microbes	Heat‐treatment (e.g., UHT, ESL, extrusion) Preservatives (e.g., sorbates) MAP, vacuum‐packing, freezing	Post‐process contamination
Spore￼forming bacteria	Partially reduced with heat‐treatment during processing	*B. cereus* , *Clostridium* spp. may survive; risk amplified by long storage
Mycotoxins	—	Not degraded by processing
Other chemical contaminants	Dilution/partial removal by washing, filtration during processing	Remaining chemicals, Packaging residues
Process contaminants	—	Acrylamide, nitrosamines, heterocyclic amines formed during heat‐treatment or cooking
Allergens	—	Cross‐contamination or concentrated by processing

Protein from raw plant material can be extracted through either dry or wet fractionation, and then further processed by using dry or high‐moisture extrusion methods. Other production methods for PBMAs are for example, fiber spinning and fermentation (Boukid et al. 2023; Thavamani et al. 2020). Both types of extrusion involve three main steps: pre‐conditioning of the raw materials before entering the extruder; heating and compression within the extruder; and cooling of the die followed by processing of the final product, such as cutting into desired pieces (Hadi and Brightwell 2021). One processing technique utilized in making PBMAs is solid‐state fermentation that has traditionally been utilized for the fermentation of various plant‐based materials. In fungi‐ and *Bacillus*‐fermented foods like tempeh, sufu, and natto, fermentation improves the protein digestibility of the plant food materials and creates texture and flavors for the products (Canoy et al. 2024).

Microbial contamination during the processing, such as the extrusion of PBMAs, can occur from contaminated manufacturing equipment (e.g., *L. monocytogenes* ) or through food handling by employees (e.g., *S. aureus* from human skin) (Banach et al. 2022). Fermentation during PBMA production may introduce additional opportunities for pathogen growth (Willis et al. 2024). Heterogeneity and batch‐to‐batch variation of the raw materials also create challenges, such as uneven microbial growth, which can be caused by nonuniform aeration and head distribution during fermentation or quality problems due to contaminating microbes, for example, spore‐forming bacteria. Essential nutrients like vitamins, iron, and long‐chain n‐3 (omega‐3) fatty acids, often missing from PBMAs, are supplemented post‐extrusion to increase the nutritional value. However, ingredients added after processing and heat treatment can be a possible microbiological risk if not sterilized before supplementation.

Microbial risk points in the processing step include insufficient heating that does not eliminate all possible harmful microbes present in the raw materials, or an improper cooling step. In addition, microbial contamination from the process equipment, process facilities, water and air, or food‐handling personnel is possible. The microbial proliferation in PBMAs can be faster than in meat‐containing counterparts, especially if not properly cooled (Tóth et al. 2021). In addition, high pH and water activity values in many PBMAs can support microbial growth (Willis et al. 2024).

Mitigation strategies to eliminate or decrease the microbiological risks are a heating step during processing, good hygiene practices, and sufficient time–temperature management during and after the manufacturing process. Some PBMA products do not involve a heating stage during production and are thus more prone to microbial spoilage and pathogen contamination (Willis et al. 2024).

In fermentation, typically used lactic acid bacteria (LAB) lower the pH and extend the shelf‐life of PBMAs by inhibiting the growth of foodborne pathogenic bacteria and food‐spoiling microbes (Boukid et al. 2023). The predominance of members of the LAB group in PBMA may be relevant for the product stability. In conventional fermented meat products, LAB and starter cultures (incl. *Staphylococcus carnosus* and *Staphylococcus xylosus* ) can accelerate the degradation of proteins and fats to produce flavor compounds, enhance palatability, develop compact meat quality, and extend shelf‐life by inhibiting the growth of food‐borne pathogenic bacteria such as *E. coli* and *Enterobacteriaceae*.

Many PBMAs are frozen after manufacturing to extend their shelf‐life, and refrigerated products are often packaged in modified atmosphere packaging (MAP) or vacuum‐packed to prevent the growth of aerobic spoilage bacteria and molds (Grove 2022). Chilled ready‐to‐eat products are high‐risk products, and especially *Listeria* and *Clostridium* species can be a concern in these types of products (Banach et al. 2022).

In contrast to meat products, nitrite is not an allowed food additive in PBMAs, which belong to category 12.9 in the Regulation (EC) No 1333/2008. In this category, only the following preservatives are allowed: sorbates E200‐E202 (analogues of meat, fish, seafood and cheese) and sulfites E220‐228 (analogues of meat, fish and seafood). Potassium sorbate primarily works by disrupting microbial cell membranes and inhibiting enzymes essential for energy production. Its antimicrobial activity depends on pH, with peak effectiveness at pH values below 6.5. While sorbates are most potent against yeasts and molds, they also inhibit certain bacteria, including vegetative cells of *B. cereus* , non‐proteolytic *C. botulinum* and *L. monocytogenes* (Melin 2024; Smoot and Pierson 1981). In acidic conditions, potassium sorbate has proven effective in preventing the germination of *C. botulinum* and *B. cereus* spores (Smoot and Pierson 1981). Their effectiveness is enhanced when used in combination with other preservation strategies, such as high‐pressure processing (HPP) or mild heat treatments like pressure‐assisted thermal processing (PATP) (Astorga‐Oquendo et al. 2024).

Overall, processing plays an important role in shaping the safety profile of PBDAs and PBMAs. Thermal treatments such as UHT processing, ESL treatment, and extrusion are generally effective in reducing vegetative pathogens, yet they have limited impact on pre‐existing chemical hazards such as mycotoxins and heavy metals, which largely reflect raw material quality (Table 7). In addition, heat‐resistant spore‐forming bacteria may survive processing and pose a risk during extended storage, particularly in products with high water activity and near neutral pH. Post‐processing contamination remains a critical vulnerability for both PBDAs and PBMAs, underscoring the importance of hygienic design, environmental monitoring, and process validation. Compared with PBDAs, PBMAs present a more complex microbiological risk profile due to milder heat treatments, diverse ingredient matrices, and the absence of nitrites, which necessitates alternative hurdle strategies. Together, these findings highlight that while many hazards are effectively controlled by current processing technologies, others persist, requiring targeted control measures and continued surveillance to ensure product safety.

## 8 Conclusions

This review highlights the significant food safety concerns associated with plant‐based dairy and meat alternatives. While these products offer numerous nutritional benefits and cater to the growing demand for sustainable and ethical food choices, they also present unique challenges in terms of chemical and microbiological hazards.

Raw agricultural ingredients used in PBDA and PBMA may introduce hazards that differ from those typically found in milk and meat. These include various mycotoxins and certain spore‐forming microbes, although the latter can also be present in meat. Although quality control of the raw materials and the processing eliminates some of the hazards, they have also been identified in the final products. Products made from different plants have partly different contaminant profiles.

The so‐called emerging mycotoxins such as enniatins and Alternaria toxins are a group of chemical hazards that may need to be monitored and controlled in the future, as literature shows they are present in most plant‐based drink types and also appear to be widely spread in meat alternatives. Thus, replacement of animal‐based products in the diet can increase the consumer's exposure to many of the emerging mycotoxins. These compounds are not yet controlled in any food types, and toxicological data on the health effects of some of them are still scarce. Assessing the risk of harmful health effects from exposure to the emerging mycotoxins will be necessary in the future, and PBDAs and PBMAs should be included in the assessment as potential important sources.

Drinks can be a large source of inorganic arsenic, and only rice‐based drinks have currently maximum levels set for inorganic arsenic content in the EU legislation. The literature reviewed here indicates that nut‐based drinks are also a potential source of elevated inorganic arsenic, although the data are still scarce. Risk management actions may be needed, but more data on the occurrence are required before decision making.

According to limited data (one study each), bisphenol A, atropine, and heterocyclic aromatic amines in PBDAs are also a potential risk, but more occurrence data are also needed for these compounds, which are not currently monitored in drinks.

The literature also indicates that aluminium, cadmium, nickel, and lead concentrations in some PBDA types can be elevated, and high PBDA consumption could increase the consumers' exposure from the whole diet to the extent that the risk of harmful health effects cannot be ruled out. Nickel and lead in soy‐based PBMAs should also be studied more, as the literature indicates their concentrations can be higher than in animal‐based alternatives.

PBMAs are also a potential source of process contaminants. The concentration of acrylamide in PBMA products varies, and in some products the levels are relatively high. Consequently, individuals who consume these products frequently may have a higher exposure to acrylamide. However, the occurrence of acrylamide in PBMA has been investigated only to a limited extent, and additional research and risk assessment are needed.

Certain food additives are allowed in PBMAs that are not allowed in the corresponding animal‐based foods, and vice versa. Therefore, dietary change to a more plant‐based diet can also increase the intake of some food additives while decreasing that of others.

PBDAs and PBMAs are formulated using similar high‐moisture ingredients rich in nutrients and possessing near‐neutral pH levels, which altogether create highly favorable conditions for microbial proliferation. Under improper handling or storage, both categories can support the growth of spoilage organisms and foodborne pathogens. However, the specific microbiological risks and dynamics differ considerably due to variations in processing methods and storage conditions.

Nevertheless, surveillance studies indicate that microbiological contamination of PBDAs is infrequent, although the sample sizes in these studies have generally been limited. The majority of commercially available products appear to undergo sufficient heat treatment to ensure safety. Among the microorganisms detected, *B. cereus* appears to be the most common potentially pathogenic species, likely due to its ability to form heat‐resistant spores. Despite the low prevalence of pathogens, foodborne outbreaks linked to PBDAs demonstrate that, when contamination occurs, the intrinsic properties of these products can facilitate pathogen growth and pose a health risk. Additionally, extended shelf life may further contribute to the potential for pathogen proliferation during storage.

In contrast, PBMAs exhibit a more complex microbiological risk profile. The manufacturing processes often involving extrusion and milder heat treatments are designed to impart desirable texture and sensory properties but may not fully inactivate resilient microorganisms. Research data from various European countries demonstrated a wide range in total viable counts and identified the presence of spoilage organisms and occasional pathogens in PBMAs including *L. monocytogenes* , *S. aureus* , *E. coli* , and *Salmonella*. Additionally, spore‐forming bacteria such as *B. cereus* and *Clostridium* species can be present. They can survive thermal processing and pose a risk during storage and distribution. Notably, the occurrence of *C. botulinum* in vegetarian sausages at a reported prevalence of 32% underlines the risk associated with vacuum packaging and mild thermal processing. In meat products, the risk of *C. botulinum* is mitigated also via use of nitrites as food additives, but although the current EU legislation allows some preservatives in PBMAs, the addition of nitrites is not allowed in these products. As product varieties and processing technologies expand, safety assessments must keep pace. Future needs include development of PBMA‐specific thermal and non‐thermal inactivation technologies, broader surveillance of emerging mycotoxins in both raw materials and finished products, improved hygienic design and monitoring to prevent post‐processing contamination, strategies to control *C. botulinum* and *B. cereus* in the absence of nitrites, and evaluation of process‐induced contaminants in novel protein matrices.

Overall, while most PBDA and PBMA products on the market demonstrate satisfactory microbial safety, the presence of heat‐resistant spore‐formers and the potential for post‐processing contamination necessitate a robust food safety strategy. This includes strict hygienic processing conditions, process validation for microbial inactivation, continuous surveillance, and consumer guidance on proper storage and handling. More research is needed to identify potential safety and health concerns associated with the new generation of PBMAs, as well as to develop technological innovations to mitigate any potentially adverse effects. The food safety of PBDA and PBMA is also influenced by consumer behavior, as the spoilage of a product is often not as easily detectable by consumers' sensory assessment as, for example, the acidification of milk. Preliminary results from our ongoing study (www.ruokavirasto.fi/en/vegeturva) suggest that some consumers store PBDA in a different way than similar dairy products.

European Food Safety Authority EFSA has also identified a need for more data on the prevalence of microbiological hazards in ready‐to‐eat PBDAs and PBMAs and has funded a prevalence study covering 15 EU Member States. This study, led by the Food Safety Authority of Ireland, ended in early 2026 and will likely be published by the end of 2026.

There are nutritional differences between dairy products and PBDAs, the magnitude of which varies between PBDA types. In addition, the absorption of nutrients may be different from dairy products. Many PBDAs contain dietary fiber, which is not present in milk, and PBDAs do not contain lactose or cholesterol. Fatty acid compositions vary widely between product types, but with the exception of coconut drink, most PBDAs contain more polyunsaturated fatty acids than milk. Cereal‐based products are lower in protein than milk, which can be detrimental to sensitive populations, and non‐fortified products are not recommended for infants due to the risk of vitamin deficiency. Soy products can also have a slight estrogenic potential, the health effects of which are insufficiently understood.

Likewise, the protein content of PBMAs may be lower than in comparable meat products, and fortification by essential nutrients such as iron and vitamin B12 is necessary to ensure adequate intake. On the other hand, PBMAs contain more fiber than meat and often less saturated fat, which increases their nutritional quality.

To our knowledge, only a limited number of risk assessments have been conducted on PBDAs and PBMAs. While this review does not aim to perform a risk assessment, the data compiled here provide information on the concentrations of various hazards in these products at retail level, which represent potential exposure amounts for consumers. Such information can serve as a valuable basis for future risk assessment studies. The need for comprehensive risk assessments of PBDAs and PBMAs is growing, as these products are increasingly incorporated into plant‐based diets. A study by Valsta et al. (2025) reports that transitioning to a plant‐based diet can improve the overall nutritional quality and reduce climate impact; however, it may slightly increase exposure to certain chemical hazards, such as metals and aflatoxins. In addition, the intake of some nutrients—such as vitamins A, D, B1, B2, and B12, as well as iron and folate in women—may become insufficient in a predominantly plant‐based diet. Understanding these risks is critical to safeguard food safety and ensure nutritional adequacy as dietary patterns continue to shift.

Despite these challenges, plant‐based dairy and meat alternatives hold promise for addressing global food security and environmental sustainability. Continued research and innovation are essential to improve the safety and quality of these products. Collaboration between industry stakeholders, regulatory bodies, and researchers will play a pivotal role in developing effective risk management strategies and ensuring the safe consumption of plant‐based foods.

## Author Contributions

**Petra Pasonen:** conceptualization, investigation, funding acquisition, writing – original draft, writing – review and editing, methodology, visualization. **Elina Sohlberg:** writing – original draft, writing – review and editing, investigation, methodology, funding acquisition, conceptualization, visualization. **Hanna‐Leena Alakomi:** conceptualization, investigation, funding acquisition, writing – original draft, writing – review and editing, methodology. **Johanna Suomi:** project administration, conceptualization, investigation, funding acquisition, writing – original draft, writing – review and editing, methodology.

## Funding

This manuscript has been prepared as part of research project “VEGETURVA” (www.ruokavirasto.fi/en/vegeturva) funded by the Finnish Ministry of Agriculture and Forestry with contribution from Valio Ltd., Raisio Oyj and Fazer Group (Makera funding, decision VN/10341/2022).

## Data Availability

Data sharing not applicable to this article as no datasets were generated or analyzed during the current study.
